# Evaluation of e-liquid toxicity using an open-source high-throughput screening assay

**DOI:** 10.1371/journal.pbio.2003904

**Published:** 2018-03-27

**Authors:** M. Flori Sassano, Eric S. Davis, James E. Keating, Bryan T. Zorn, Tavleen K. Kochar, Matthew C. Wolfgang, Gary L. Glish, Robert Tarran

**Affiliations:** 1 Marsico Lung Institute/Cystic Fibrosis Research Center, University of North Carolina at Chapel Hill, Chapel Hill, North Carolina; 2 Department of Chemistry, University of North Carolina at Chapel Hill, Chapel Hill, North Carolina; 3 Department of Microbiology and Immunology, University of North Carolina at Chapel Hill, Chapel Hill, North Carolina; 4 Department of Cell Biology & Physiology, University of North Carolina at Chapel Hill, Chapel Hill, North Carolina; Stanford University, United States of America

## Abstract

The e-liquids used in electronic cigarettes (E-cigs) consist of propylene glycol (PG), vegetable glycerin (VG), nicotine, and chemical additives for flavoring. There are currently over 7,700 e-liquid flavors available, and while some have been tested for toxicity in the laboratory, most have not. Here, we developed a 3-phase, 384-well, plate-based, high-throughput screening (HTS) assay to rapidly triage and validate the toxicity of multiple e-liquids. Our data demonstrated that the PG/VG vehicle adversely affected cell viability and that a large number of e-liquids were more toxic than PG/VG. We also performed gas chromatography–mass spectrometry (GC-MS) analysis on all tested e-liquids. Subsequent nonmetric multidimensional scaling (NMDS) analysis revealed that e-liquids are an extremely heterogeneous group. Furthermore, these data indicated that (i) the more chemicals contained in an e-liquid, the more toxic it was likely to be and (ii) the presence of vanillin was associated with higher toxicity values. Further analysis of common constituents by electron ionization revealed that the concentration of cinnamaldehyde and vanillin, but not triacetin, correlated with toxicity. We have also developed a publicly available searchable website (www.eliquidinfo.org). Given the large numbers of available e-liquids, this website will serve as a resource to facilitate dissemination of this information. Our data suggest that an HTS approach to evaluate the toxicity of multiple e-liquids is feasible. Such an approach may serve as a roadmap to enable bodies such as the Food and Drug Administration (FDA) to better regulate e-liquid composition.

## Author summary

The e-liquids used in electronic cigarettes (E-cigs) typically consist of a mixture of propylene glycol (PG), vegetable glycerin (VG), and nicotine, as well as numerous chemical additives that are used for flavoring. There are currently over 7,700 different flavored e-liquids that are commercially available, but there is very limited information regarding either their chemical composition or toxicity. In this work, we developed a high-throughput screening (HTS) assay to rapidly triage and validate the toxicity of multiple e-liquids in parallel. Our data indicated that e-liquids are extremely heterogeneous, so we also performed gas chromatography–mass spectrometry (GC-MS) of all e-liquids to evaluate their composition/toxicity relationship. We found that the presence of either vanillin or cinnamaldehyde in e-liquids was associated with higher toxicity values. In addition, our data demonstrated that the PG/VG vehicle by itself was toxic at higher doses. We have also developed a publicly available and searchable website (www.eliquidinfo.org) that contains these chemical composition and toxicity data. Given the large numbers of available e-liquids, this website will serve as a resource to disseminate this information. Our HTS approach may serve as a roadmap to enable bodies such as the United States Food and Drug Administration (FDA) to better regulate e-liquid safety.

## Introduction

Electronic cigarettes (E-cigs), also known as electronic nicotine delivery systems (ENDS), are devices that deliver nicotine to the lung without combustion in a process known as “vaping” [1]. They differ from traditional cigarettes in that they do not contain tobacco, and—instead—they produce an aerosol by drawing and heating a liquid vehicle (e-liquid) over a battery-powered coil. This aerosol is inhaled and deposited in the lungs so that nicotine can be absorbed into the bloodstream and translocate to the brain [2]. E-cigs were introduced as a potentially safer alternative to tobacco smoking because they do not contain the toxic byproducts of tobacco combustion, including tar-phase chemicals [3, 4]. However, vaped e-liquids also undergo pyrolysis and generate oxidative species, which may lead to the formation of additional toxic components (i.e., formaldehyde and carbonyls) that are similar to those seen in cigarettes [5, 6]. In addition, while e-liquids do not contain tobacco, they may contain nicotine derived from tobacco and therefore may contain certain tobacco-related components such as nitrosamines [7]. However, despite these observations, little is known about the toxicity potential of most e-liquids. Since their inception, E-cig design has progressed rapidly. The first-generation E-cigs, dubbed “cigalikes,” were prefilled disposable devices that were designed to look like traditional cigarettes. In contrast, second- and third-generation E-cigs have interchangeable parts including an aerosol generator, a heating element (coil), a refillable tank, and much more powerful rechargeable batteries [8, 9]. These devices have broken from the traditional design in favor of handheld tanks that have an increased and even customizable ability to deliver aerosolized nicotine (along with other aerosolized constituents) [10]. Moreover, second- and/or third-generation E-cigs produce a higher concentration of plasma nicotine metabolites (cotinine and trans-3’-hydroxycotinine) than the first-generation cigalikes that is now comparable to plasma cotinine levels seen in regular smokers [11, 12].

The e-liquid vehicle used in E-cigs is composed of propylene glycol (PG) and vegetable glycerin (VG) at varying ratios. There are currently over 7,700 e-liquid flavors on the market from over 1,200 different vendors in the United States, and the number continues to increase [10]. E-liquids come in many different flavors, colors, nicotine concentrations (0–36 mg/mL) and PG/VG ratios (e.g., 80:20, 70:30, 55:45, and 40:60). Despite their ubiquity, manufacturing standards for e-liquids do not currently exist, and they can differ in composition from vendor to vendor [13].

The sheer diversity and variability have made it difficult to comprehensively study e-liquids, and to date, very little to no research has been conducted to assess the safety of most available e-liquids. Many of the chemical constituents in e-liquids, including PG and VG, are on the Food and Drug Administration (FDA)’s Generally Recognized As Safe (GRAS) list. However, most GRAS studies on flavors were performed following oral ingestion in rats [14, 15], and many GRAS chemicals have not been tested for safety after inhalation [16–18]. Indeed, the toxicity profile for inhalation is markedly different from the oral route. As a case in point, diacetyl, which is used as butter-flavored chemical, is on the GRAS list but causes bronchiolitis obliterans when inhaled [19, 20]. Emerging studies have shown that e-liquids have measurable biological effects on cells, including altering Ca^2+^ signaling, cell growth, viability, and inflammation. However, the research that has been conducted thus far has looked at only a small proportion of the available e-liquids, leaving the effects of many e-liquid flavors unknown [21–24]. Given the growing number of untested, commercially available e-liquids, new paradigms need to be introduced to rapidly screen these e-liquids using in vitro assays to better inform both the policy makers (i.e., the legislature/FDA) as well as the public. Here, we introduce a high-throughput screening (HTS) assay designed to assess growth characteristics, viability, and chemical composition of e-liquids. The overall goal of this work was to screen neat e-liquids and identify potential flavors and/or chemical constituents that were more toxic than PG/VG and would warrant additional, more detailed attention. Therefore, as a proof of concept, we screened 148 e-liquid flavors to determine their relative toxicity and chemical composition. We then validated these results in multiple cell types and after exposure to E-cig aerosols.

## Results

### Initial screen to assess e-liquid in vitro toxicity

**Fig 1:**
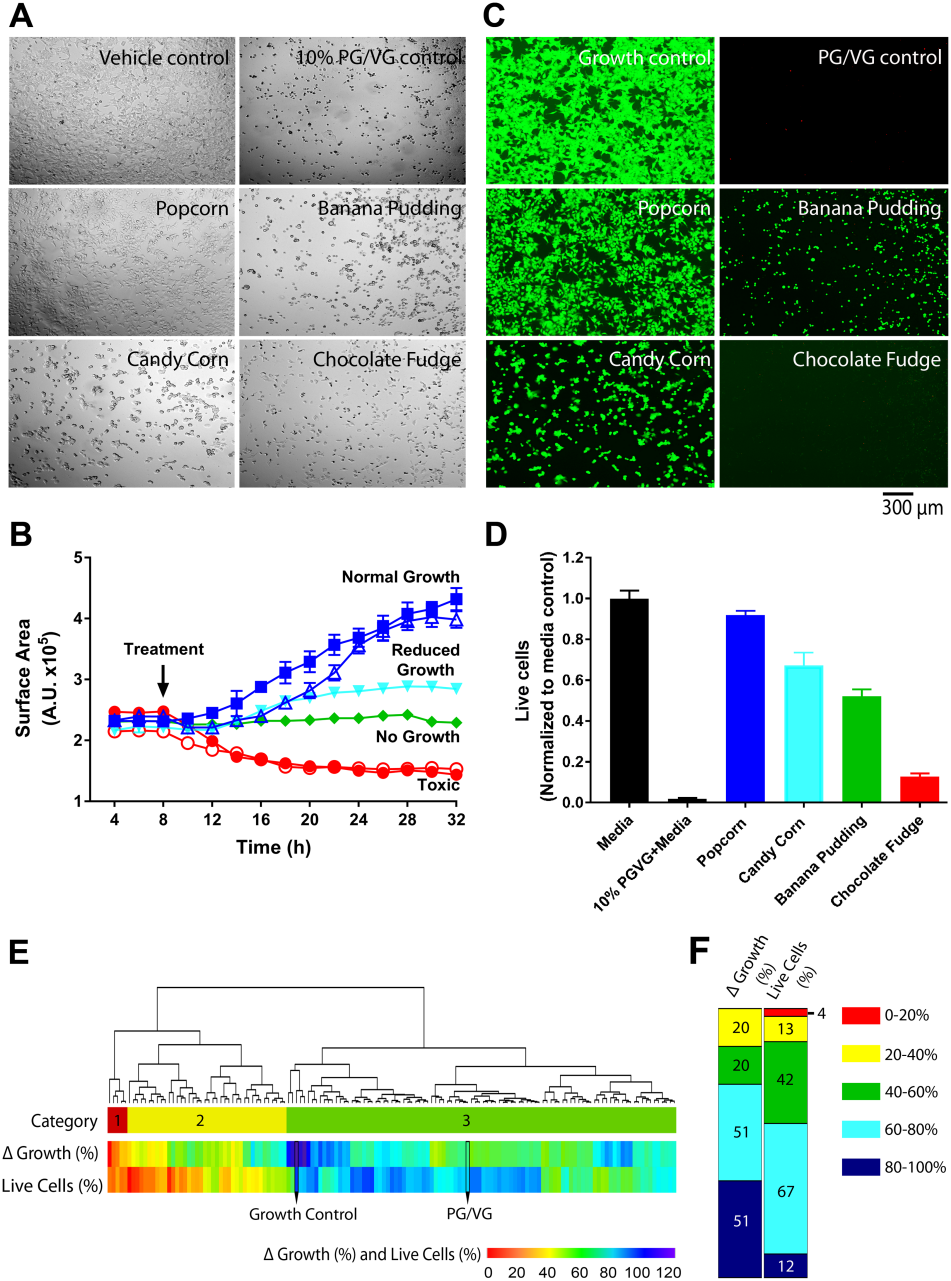
Development of preliminary screens to assess e-liquid toxicity in vitro. Cells were incubated for 8 h in 384-well plates, e-liquids were added as indicated for 24 h, and bright-field images were automatically obtained every 2 h to determine growth rates. Cell surface area, as an indicator of confluency, was normalized to the media control. All *N =* 4. (A) Bright-field images of HEK293T cells incubated overnight with vehicle, 10% PG/VG + media, or 1% banana pudding-, candy corn-, chocolate fudge-, or popcorn-flavored e-liquids. (B) Mean representative growth curves obtained from the bright-field images over time. Curves were categorized as follows: normal growth, media control (■) and popcorn (Δ); reduced growth, candy corn (▲); no growth, banana pudding (♦); and toxic, 10% PG/VG (●) and chocolate fudge (○). (C) Images of HEK293T cells stained with calcein-AM after overnight incubation with vehicle, 10% PG/VG + media, 1% banana pudding-, candy corn-, chocolate fudge-, or popcorn-flavored e-liquids. (D) Quantification of calcein-AM fluorescence (i.e., viability) expressed as mean ± SEM. All *n =* 3. The positive control (10% PG/VG + media) used the same PG/VG ratio as the e-liquids. (E) Heat map depicting Δ growth (%) and live-cell fluorescence (%). Growth control and PG/VG controls are shown for reference. E-liquids are grouped in 3 distinct categories from the clustering: 1 (red), e-liquids that showed low Δ growth and live-cell fluorescence % (0%–40%); 2 (yellow), e-liquids that showed moderate (40%–100%) Δ growth and low live-cell fluorescence % (0%–40%); and 3 (green), e-liquids that showed high Δ growth and live-cell fluorescence % (80%–100%). (F) E-liquids were grouped according to Δ growth and live-cell fluorescence. Numbers represent number of e-liquids in a category. Raw data are available in S1 Data. HEK293T, human embryonic kidney 293 cells; PG, propylene glycol; VG, vegetable glycerin.

We initially designed 2 screens to assess cellular toxicity. The first method consisted of quantifying cell surface area by thresholding automatically acquired bright-field images over time as an indicator of cell growth. Using this approach, we assessed the effects of 148 e-liquids and a PG/VG control (added at 1% and 10%, respectively) to human embryonic kidney 293 (HEK293T) cells cultured in 384-well plates. Cells were plated at a density of 5,000 per well and placed in an imaging plate reader for 8 h at 37 °C, 5% CO_2_. After addition of the vehicle control (100% media), cells exhibited normal, log-phase growth over 12 to 32 h and showed duplication of cell surface area, consistent with healthy cell growth (Fig 1A and 1B; S1 Data). Addition of 10% 55:45 PG/VG in media significantly attenuated cell growth, which served as a negative control in subsequent studies. Fig 1A (S1 Data) depicts representative images from cells exposed to different e-liquids (Popcorn, 88% Δ growth; Candy Corn, 86% Δ growth; Banana Pudding, 18% Δ growth; Chocolate Fudge, 14% Δ growth), as well as phosphate-buffered saline (PBS) and PG/VG controls. We classified the complete growth curves for these e-liquids as normal, reduced, no growth, and toxic (Fig 1B; S1 Data). The second approach used to assess toxicity of e-liquids was to fluorescently measure the number of live cells using calcein-AM (Fig 1C; S1 Data). Using this approach, we detected significant attenuation of viability (i.e., decreases in calcein fluorescence) after 24 h (see Fig 1D for representative examples and Fig 1E and 1F for summary data; S1 Data). We then performed hierarchical clustering on all e-liquids tested, taking into account both Δ growth and live-cell fluorescence (Fig 1E; S1 Data). Using complete agglomerative hierarchical clustering, e-liquids could be separated into 3 relevant categories: (i) red, e-liquids that showed low Δ growth and low live-cell fluorescence; (ii) yellow, e-liquids that showed moderate Δ growth and low live-cell fluorescence; and (iii) green, e-liquids that showed higher Δ growth and high live-cell fluorescence. Because we could discern distinct trends based on the clustering methods, we then compared e-liquids according to their growth rates and viability, and we found that the fluorescence assay—which meets these criteria—was more sensitive than the cell growth density (Fig 1F; S1 Data). The coefficient of variation for this method, which indicates the variation of a standard measurement throughout a 384-well plate, was below 15% (≤20% is considered satisfactory). In addition, the signal-to-background ratio was 3.47, indicating a significant degree of separation between them. Finally, we calculated the Z’ score to quantify the suitability of this assay for use in high-throughput screens and found it to be 0.84. An assay with a Z’ score between 0.5 and 1.0 is considered an excellent assay because the separation between the positive and negative controls, relative to the variability, is significant [25].

### PG/VG alone affects cell viability

**Fig 2:**
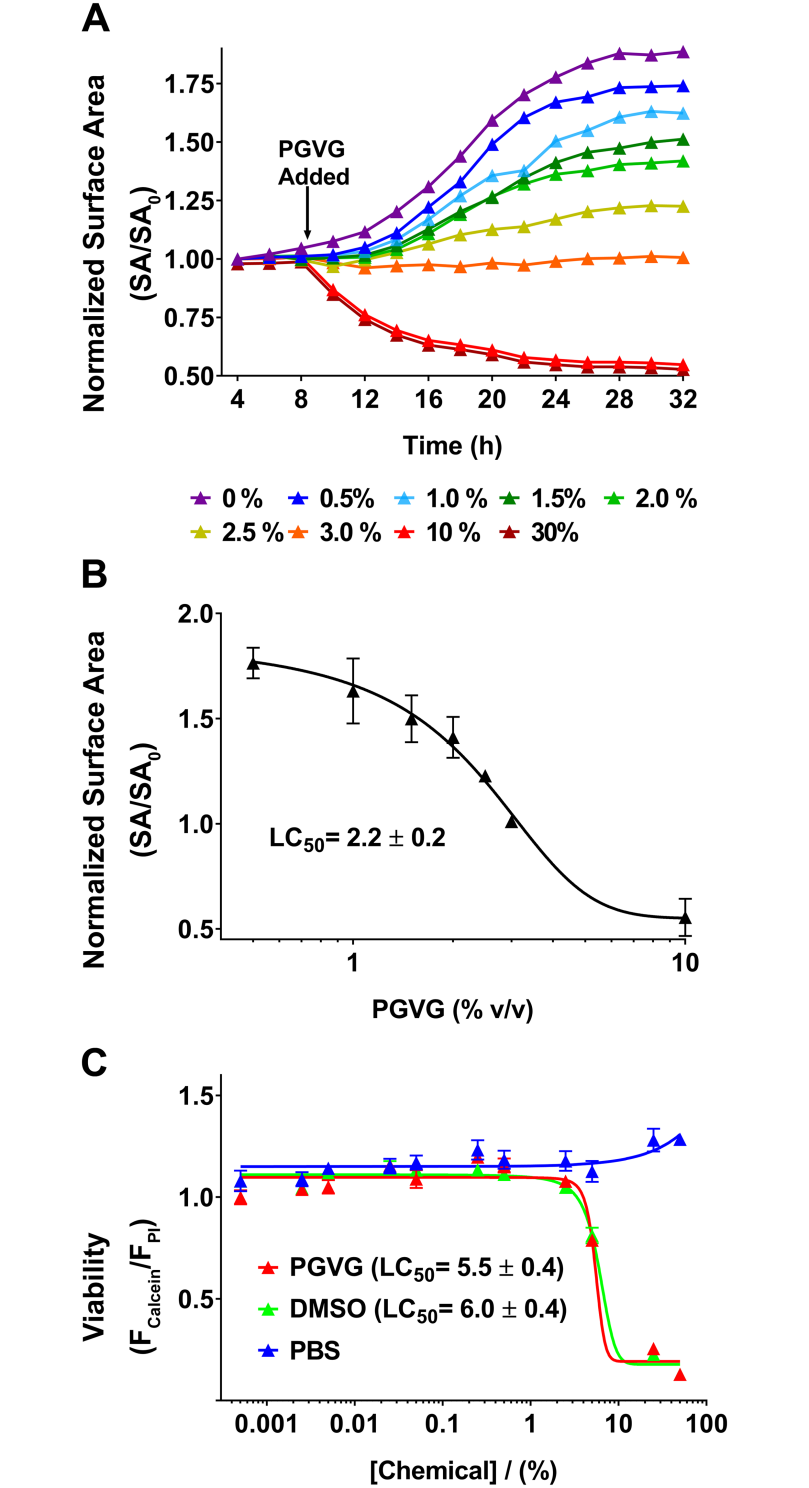
PG/VG alone negatively affects cell viability. (A) Several concentrations of 55:45 PG/VG (0%–30% range) were added to HEK293T and incubated for 24 h. Cell viability was assessed via surface area using bright-field images (*N =* 3). (B) PG/VG alters cell growth in a dose-dependent manner. LC_50_ = 2.2 ± 0.2% (*N =* 4). (C). Viability was calculated measuring the ratio of fluorescence of calcein-AM/propidium iodide. PBS (nontoxic growth control), DMSO (toxic control), and 55:45 PG/VG were added to cells and incubated overnight. DMSO and PG/VG show similar LC_50_ values (*N =* 3, *p* = 0.68), while PBS was significantly different (*p* < 0.0001). Raw data are available in S2 Data. DMSO, dimethyl sulfoxide; HEK293T, human embryonic kidney 293 cells; LC_50_, concentration at which a given agent is lethal to 50% of the cells; PBS, phosphate-buffered saline; PG, propylene glycol; VG, vegetable glycerin.

Because PG/VG is an integral component of all commercially available e-liquids and appeared to induce toxicity (Fig 1C and 1D; S1 Data), we then studied its effects on cell toxicity alone by performing dose–response curves for 55:45 PG/VG. Because live-cell fluorescence was more sensitive than cell growth (Fig 1F; S1 Data), we extended this assay and simultaneously measured calcein and propidium iodide as markers or live and dead cells, respectively, as described for tobacco exposure [26]. Here, we used dimethyl sulfoxide (DMSO) as a known toxic control [27] and PBS as a nontoxic control. Serial dilutions in DMSO resulted in a decrease in cell viability with an LC_50_ (i.e., the concentration at which a given agent was lethal to 50% of the cells) of 6.0 ± 0.4%. In contrast, serial dilutions of the media with PBS did not affect cell viability and therefore could not be fitted with the equation parameters required to calculate LC_50_. PG/VG caused dose-dependent decreases in cell growth with an LC_50_ of 2.2 ± 0.2% (Fig 2A and 2B; S2 Data). We then measured cell viability using the calcein/propidium iodide assay (Fig 2C; S2 Data). PG/VG exerted a similar toxicity as DMSO (LC_50_ = 5.5 ± 0.4%; *p* = 0.68, Fig 2C; S2 Data). To test whether higher levels of PG/VG affected cell viability by reducing media O_2_ levels, we measured the partial pressure of O_2_ (P_O2_) in the media after overnight addition of 30% PG/VG using solid-state O_2_ electrodes. pO_2_ was 20 ± 1.1% (*n* = 3) in control media and 18 ± 0.4% (*n* = 4) after addition of 30% PG/VG, suggesting that the observed changes in cell growth and/or viability were not due to reduced O_2_ levels.

### E-liquids affect cell viability in a dose-dependent manner

**Fig 3:**
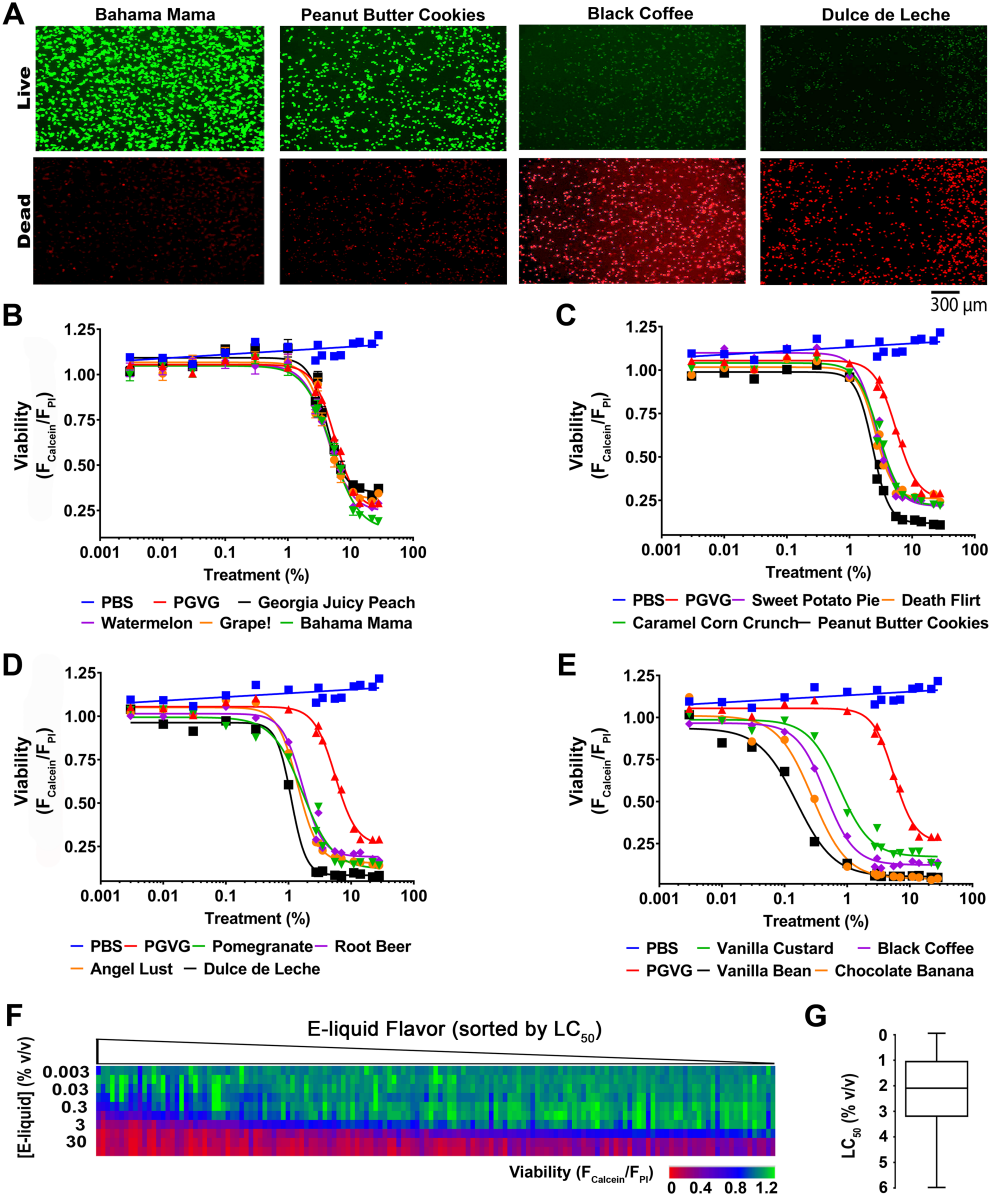
Main screen used to assess e-liquid toxicity. A total of 148 e-liquids were run as 16-point dose–response curves using the viability assay. (A) Live (calcein-AM) and dead (PI) images for representative e-liquids. (B–E) Representative e-liquid dose–response curves. PBS, negative control. PG/VG, toxic control. *N* ≥ 3. (F) Heat map of viability ratio per e-liquid, normalized to the average of the baseline. Each column represents an e-liquid flavor with increasing e-liquid (% volume/volume) and sorted by decreasing LC_50_ values. (G) LC_50_ distribution of 148 e-liquids tested (reported as % concentration). Raw data are available in S3 Data. LC_50_, concentration at which a given agent is lethal to 50% of the cells; PBS, phosphate-buffered saline; PG, propylene glycol; PI, propidium iodide; VG, vegetable glycerin.

We then generated full, 16-point dose–response curves for the e-liquids using the fluorescent viability assay (Fig 3A–3E; S3 Data). All data are shown in S1 Table. E-liquid flavors were sorted by LC_50_ values to show the range of responses (Fig 3F; S3 Data). The LC_50_ (% volume/volume) ranged from 0.14 to 6.00, and its distribution is shown in Fig 3G; S3 Data. A further summary of this data is available in an online database (www.eliquidinfo.org).

### Validation of e-liquid toxicity using human airway cell lines

**Fig 4:**
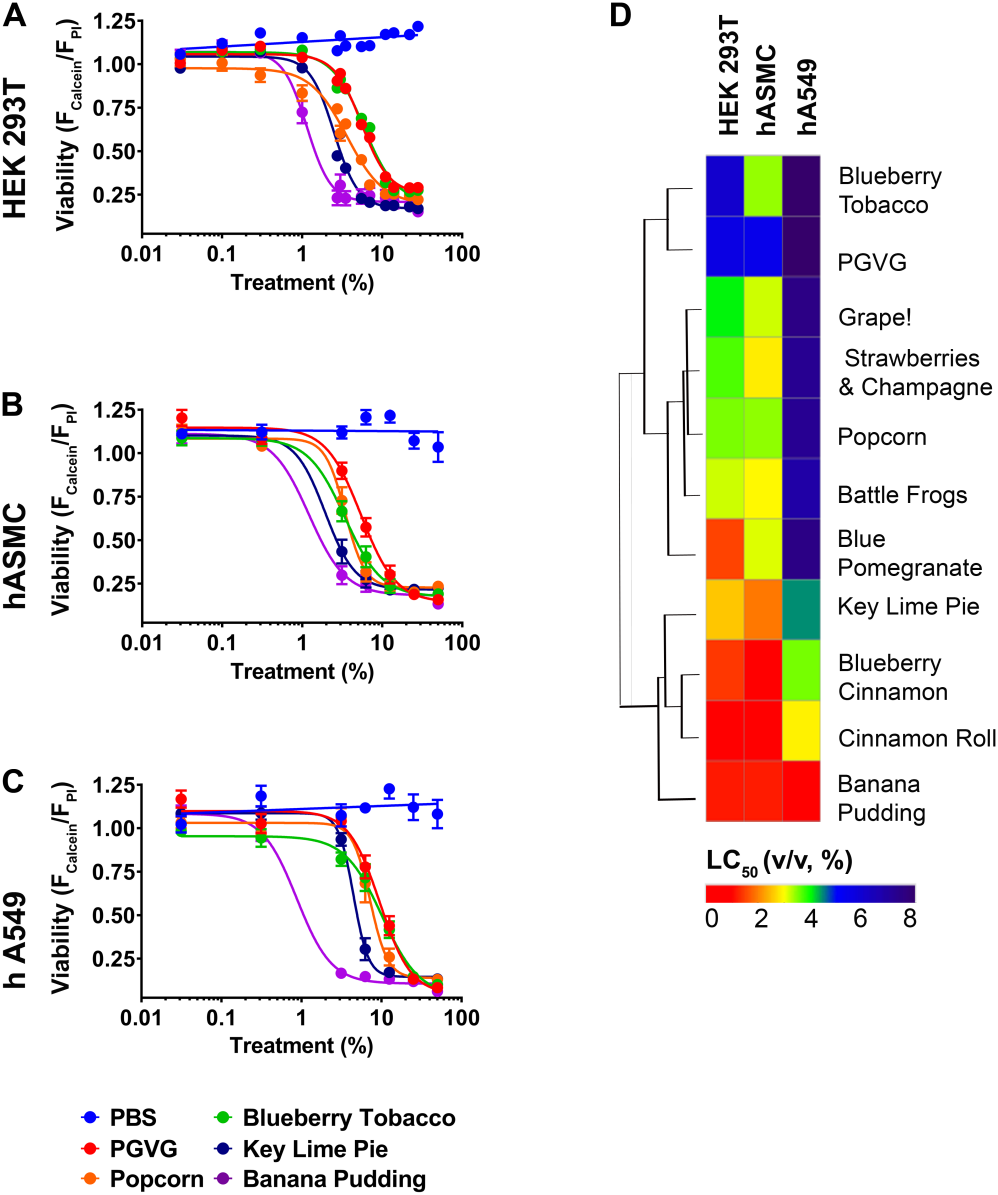
Orthogonal assays to validate human airway cell types. (A–C) Representative dose–response curves assayed in HEK293T. (A) hASMC; (B) hA549; (C) PBS (negative control), PG/VG (positive control); Blueberry Tobacco, Popcorn, Key Lime Pie, and Banana Pudding show left-shifted but similar toxicity trends. All *n =* 3. (D) Heat map showing all e-liquid flavors tested in the 3 cell lines above. E-liquids have been clustered by LC_50_ values. Raw data are available in S4 Data. hA549, human adenocarcinomic alveolar basal epithelial cells; hASMC, human airway smooth muscle cell; HEK293T, human embryonic kidney 293 cells; LC_50_, concentration at which a given agent is lethal to 50% of the cells; PBS, phosphate-buffered saline; PG, propylene glycol; VG, vegetable glycerin.

After having tested all e-liquids using HEK293T cells, we retested a subset of e-liquids in cell lines that are less suitable to HTS but more germane to the respiratory tract, namely the human adenocarcinomic alveolar basal epithelial (hA549) cell line, an immortalized cell line derived from human alveolar epithelia and primary human airway smooth muscle cells (hASMC), isolated from human large airways (see Materials and methods for details) [28, 29]. We triaged the testing by choosing every 14th e-liquid from Fig 3F (S3 Data) and generated full-dose responses for cell viability (Fig 4; S4 Data). The tested e-liquids showed a slight left curve shift for hA549 cells, indicating that these e-liquids were more toxic in these cells than in HEK293T and hASMC cells (Fig 4A–4C; S4 Data). Importantly, these e-liquids maintained the same relative toxicity in all cell lines and the LC_50_ for Banana Pudding < Key Lime Pie < Popcorn < Blueberry Tobacco (Fig 4D; S4 Data), suggesting that the use of HEK293T cells is valid.

### E-liquid aerosol exposure: Toxicity comparison

**Fig 5:**
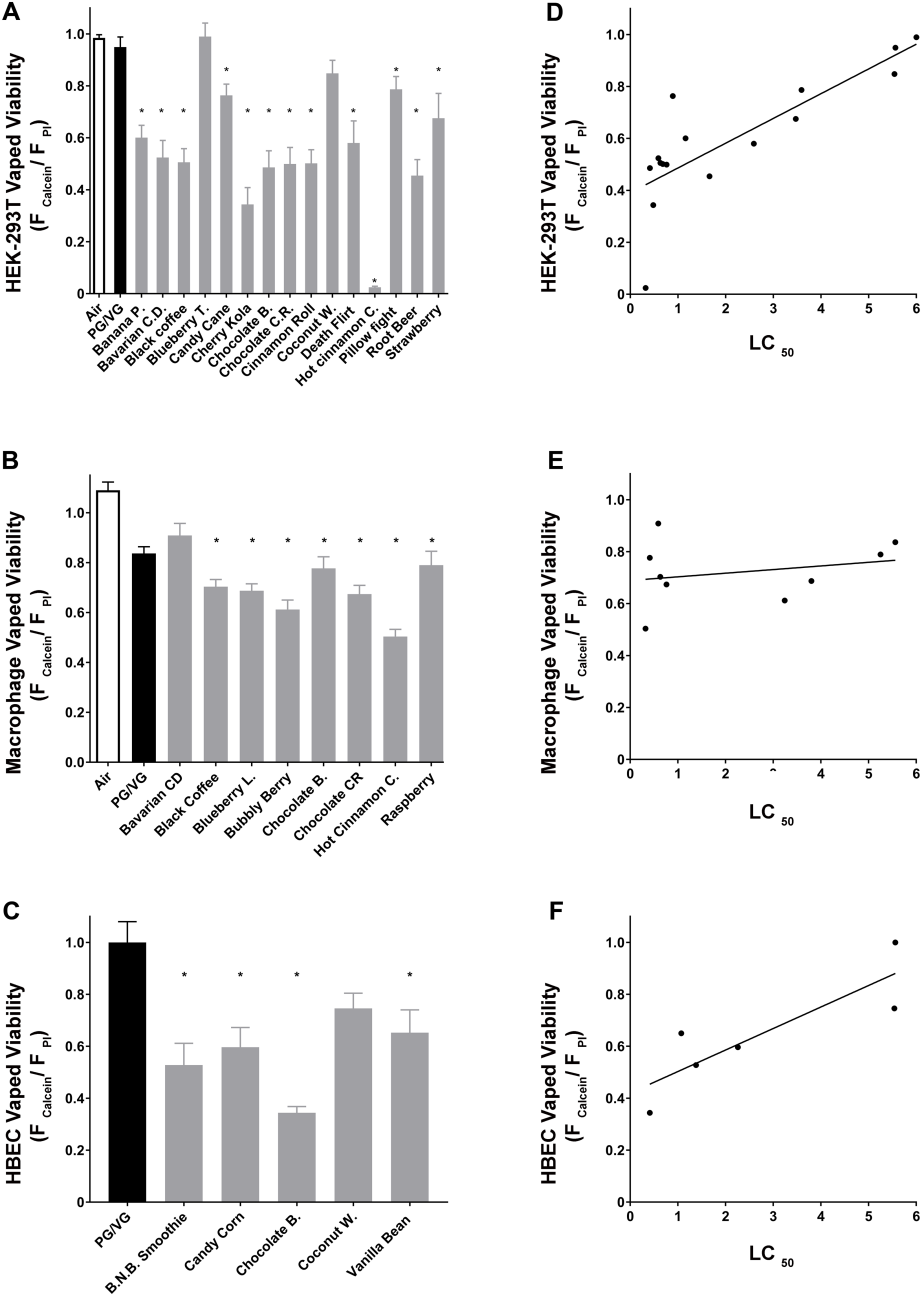
Toxicity of “vaped” versus neat e-liquids. (A) Mean normalized viability of HEK293T cells following exposure of vaped e-liquids. *N* ≥ 5 per treatment. (B) Mean normalized viability of primary human alveolar macrophages following exposure of vaped e-liquids. *N* ≥ 5 per treatment. (C) Mean normalized viability of HBECs following exposure to vaped e-liquids. *N* ≥ 5 per treatment. (D) Graph showing HEK293T vaped viability versus HEK293T toxicity (LC_50_) obtained using neat e-liquids. Linear regression R^2^ = 0.66. (E) Graph showing primary human alveolar macrophage vaped viability versus HEK293T toxicity (LC_50_). Linear regression R^2^ = 0.06. (F) HBEC viability using vaped e-liquids versus HEK293T toxicity (LC_50_). Linear regression R^2^ = 0.74. * = *p* < 0.05 different from control. For A, B, and C we performed statistical analysis using one-way ANOVA followed by Dunnett’s Test. B. N. B. Smoothie, Chocolate B., and Coconut Water. Raw data are available in S5 Data. B. N. B. Smoothie, Banana Nut Bread Smoothie; Chocolate B., Chocolate Banana; HBEC, human bronchial epithelial cells; HEK293T, human embryonic kidney 293 cells; LC_50_, concentration at which a given agent is lethal to 50% of the cells.

We have previously shown that vaped e-liquids exert similar toxicity as neat e-liquids [30]. However, during the course of vaping, e-liquids are heated to approximately 300 °C before inhalation, which may induce chemical transformations that could alter their toxicity [5]. While it is not currently possible to vape HEK293T cells under HTS conditions, we performed additional validation steps—due to the importance of this issue—by comparing the relative toxicity of e-liquids after vaping versus direct liquid addition. We selected a range of e-liquids that had high, medium, and low toxicity, as well as air and PG/VG controls. HEK293T cells and primary human macrophages were vaped in 96-well plates using a 3D printed manifold as described [30, 31]. According to our published work, 10 × 4 sec, 70 ml puffs (see Materials and methods) of e-liquid elicit significant effects on cell viability under these conditions [30]. We vaped HEK293T cells (Fig 5A; S5 Data) and primary alveolar macrophages (Fig 5B; S5 Data) using this approach. We also vaped well-differentiated human bronchial epithelial cells (HBECs) cultured at the air–liquid interface using an automated vaping system that allowed for selective exposure of HBEC mucosal surfaces to the e-liquid aerosol. Due to the larger chamber size for this system, we exposed HBECs to 70 puffs using the same puff parameters described above to achieve comparable exposures as per the HEK293T cells and macrophages. For all cell types, our data demonstrated that e-liquid vaping caused a significant decrease in viability relative to the controls that varied according to the individual e-liquids (Fig 5A–5C; S5 Data). In agreement with our previous study [30], we did not find that vaping e-liquids changed their relative toxicity.

To see if vaping correlated with direct e-liquid addition, we then plotted LC_50_ values obtained from HEK293T cells using neat e-liquids (see S1 Table) against the vaped viability (i.e., calcein/propidium iodide ratios) for the 3 different cell types (Fig 5D–5F; S5 Data). Using this approach, we observed a linear correlation for HEK293T cells (Fig 5D; R^2^ = 0.65; S5 Data) and HBECs (Fig 5F; R^2^ = 0.74; S5 Data). In contrast, vape-exposed macrophages correlated poorly to the HEK293T LC_50_ (Fig 5E; R^2^ = 0.06; S5 Data).

### Analysis of e-liquids by gas chromatography–mass spectrometry

**Fig 6:**
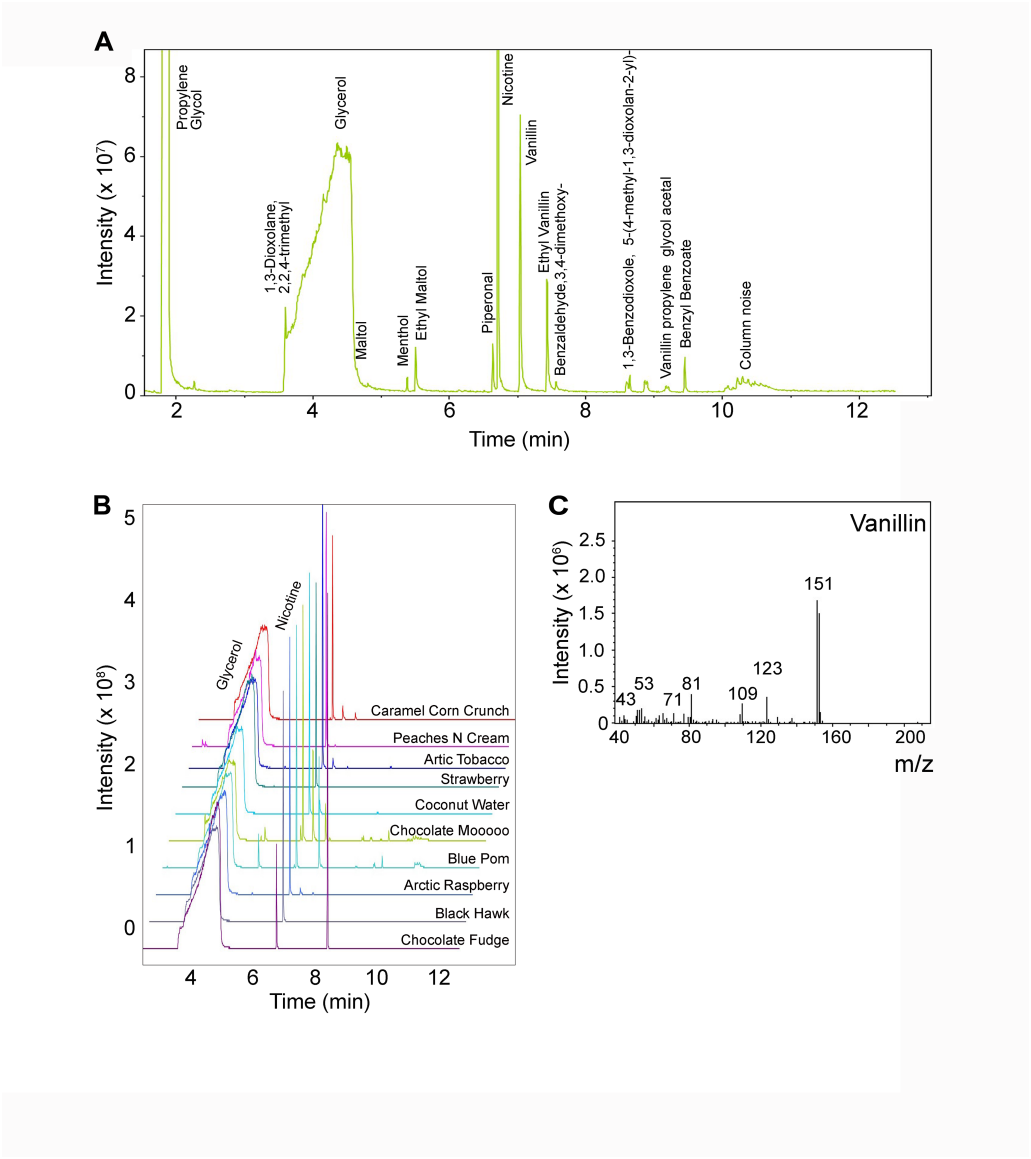
Analysis of e-liquids and their constituents by GC-MS and electron ionization. (A) Annotated chromatogram of “Dulce de Leche” e-liquid analyzed by GC-MS. PG and nicotine peaks appear off-scale to improve visibility of flavorings and additives. (B) Stacked chromatograms of 10 representative e-liquids analyzed by GC-MS. Glycerol and nicotine peaks are also shown, and PG is excluded for improved visibility of low-abundance compounds. (C) Representative electron ionization mass spectra of vanillin. GC-MS, gas chromatography–mass spectrometry; PG, propylene gycol.

In order to better understand how chemical composition contributed to e-liquid toxicity, we used gas chromatography–mass spectrometry (GC-MS) to identify e-liquid constituents. Chromatograms obtained using this approach were compared to the National Institute of Standards and Technology (NIST) 2014 mass spectral database for compound identification. Fig 6A shows a representative chromatogram of the “Dulce de Leche” e-liquid. As expected, PG, VG, and nicotine are clearly present at high concentrations. More than 10 other constituents were identified in this e-liquid, including vanillin, ethyl vanillin, and piperonal. Fig 6B depicts 10 representative chromatograms of additional e-liquids. We also performed electron ionization to obtain mass spectra to quantify select constituents present in representative e-liquids, and an example spectrum for vanillin is shown in Fig 6C.

### Bioinformatic analyses of the e-liquid population

**Fig 7:**
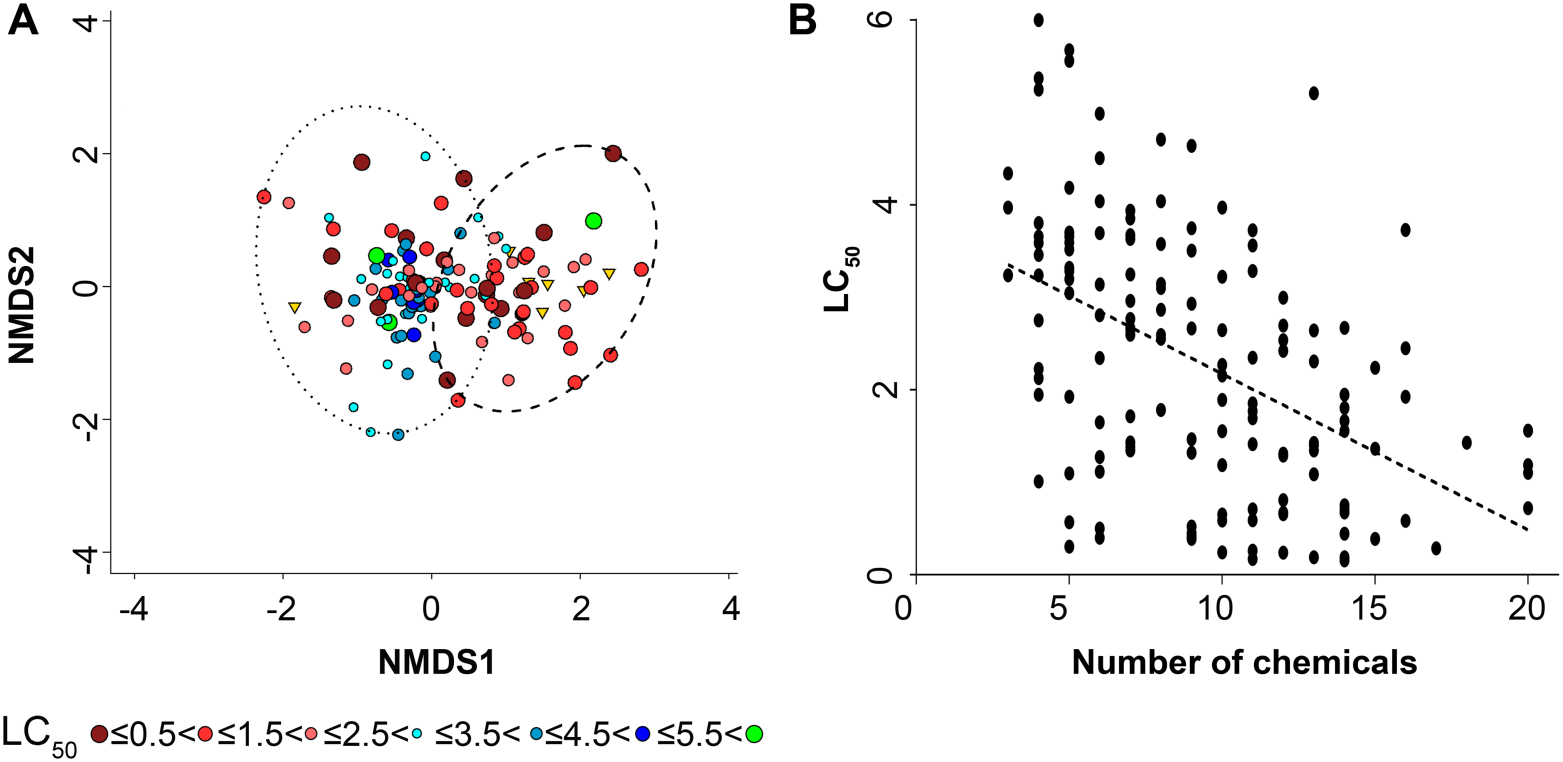
The presence/absence of e-liquid constituents and their toxicity have some correlation. (A) NMDS of e-liquids chemical analysis (presence/absence). NMDS was performed using binary Euclidean distances (stress 0.1367). E-liquids are shown as circles of varying size and color. Dashed circles encompass e-liquids within the same *k*-mode cluster, showing a significant separation of, in general, high and low LC_50_ e-liquids. Chemicals within each cluster were compared using a *t* test, in which resultant *p*-values were adjusted using Bonferroni correction. Chemicals within e-liquids are represented as triangles; gold coloration denotes those to be significantly (*p* < 0.0004) present after multiple testing correction and may be associated with lower LC_50_ values. (B) Graph showing toxicity (LC_50_) versus the number of chemicals in each e-liquid. Pearson correlation = −0.48; R^2^ = 0.23. Raw data are available in S6 Data. LC_50_, concentration at which a given agent is lethal to 50% of the cells; NMDS, nonmetric multidimensional scaling.

We compared LC_50_ values and chemical composition to identify key chemical constituents that might drive toxicity. We first compared LC_50_ values with the presence/absence of constituents to determine whether any particular chemicals and/or diversity drive the toxicity. We performed nonmetric multidimensional scaling (NMDS) on the presence/absence matrix of chemical constituents in each liquid under binary Euclidean distances, which resulted in a stress value of 0.1367, indicating a weak correlation. A qualitative separation of high and low LC_50_ values was observed that was generally supported via a *k*-modes clustering (*k* = 2). Here, a Welch two-sample *t* test showed significant differences (*p* < 0.0005) between the cluster LC_50_ means (1.55 and 2.66, respectively; Fig 7A; S6 Data). When comparing the presence/absence data of each chemical between the 2 clusters, vanillin and ethyl-vanillin—among other chemicals—were present in significantly (Bonferroni corrected; *p <* 0.0004) higher abundance (98% and 100%, respectively) in the lower LC_50_ cluster than in the higher cluster (10% and 17%, respectively). In addition, we saw a trend of negative correlation between the number of chemicals in individual e-liquids versus their toxicity (Pearson correlation = −0.48, R^2^ = 0.16) (Fig 7B; S6 Data).

**Fig 8:**
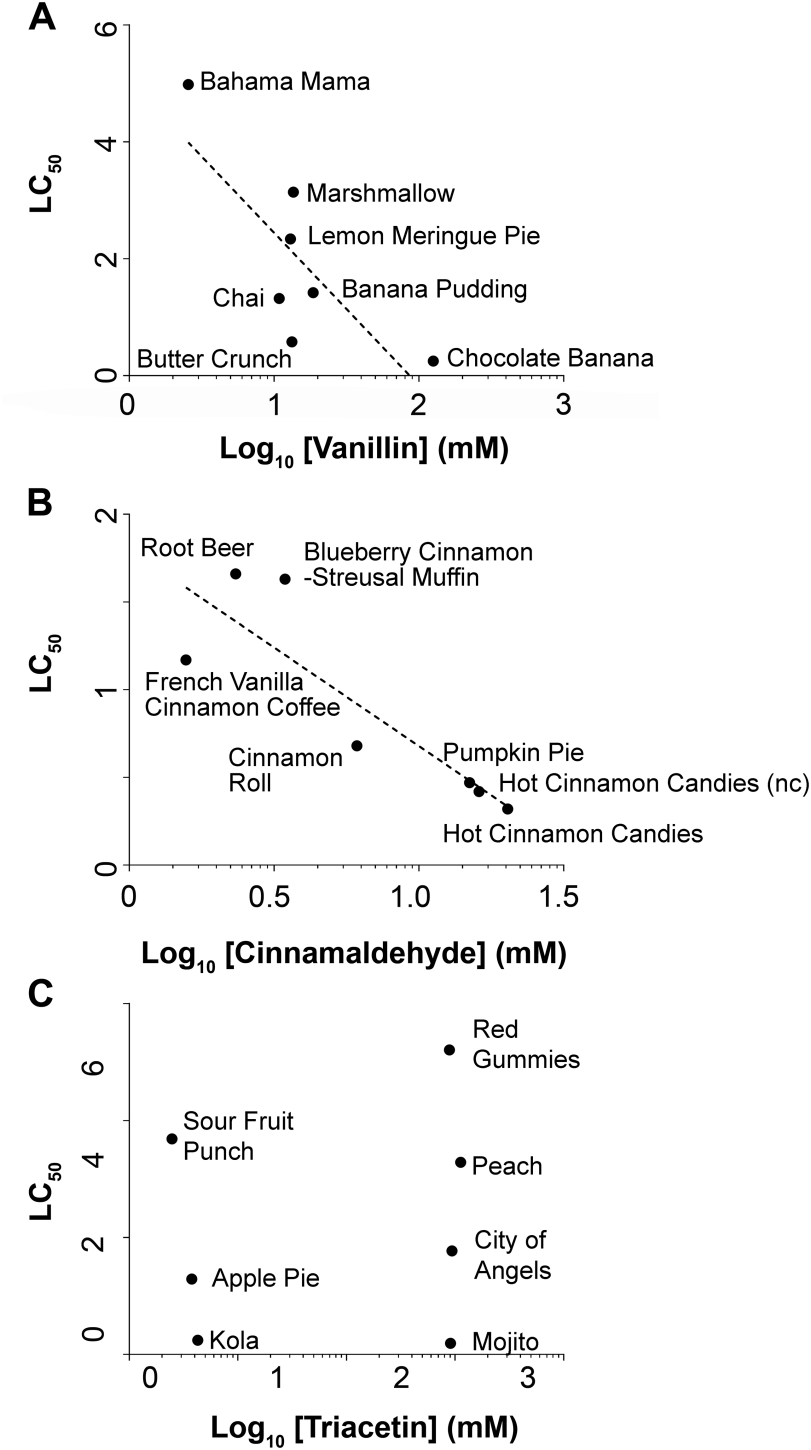
Vanillin and cinnamaldehyde concentrations correlate with toxicity in select e-liquids. (A) Graph showing toxicity (LC_50_) versus “vanillin” in select e-liquids. LC_50_ = −2.70 × log_10_(vanillin [M]) + 5.06; R^2^ = 0.62 (linear regression analysis). (B) Graph showing toxicity (LC_50_) versus “cinnamaldehyde” in select e-liquids. LC_50_ = −1.12 × log_10_(cinnamaldehyde[M]) + 1.08; R^2^ = 0.75 (linear regression analysis). (C) Graph showing toxicity (LC_50_) versus “triacetin” in select e-liquids. “N.B.” = no linear relationship was detected for triacetin. We used this chemical as an example of nontoxic control. Raw data are available in S7 Data. LC_50_, concentration at which a given agent is lethal to 50% of the cells; nc, no nicotine.

Because presence or absence of chemical constituents showed some correlation with cell toxicity, we next investigated whether the actual concentration of chemical constituents (not presence or absence) could predict toxicity. As a proof of concept, vanillin and cinnamaldehyde concentrations were measured in several e-liquids via electron ionization mass spectra because these are flavoring agents regularly used in the food industry. We also measured triacetin as a nontoxic control. We compared the concentration of each constituent and its LC_50_ values. In the case of vanillin, toxicity (i.e., LC_50_) was proportional (R^2^ = 0.62) to the actual vanillin concentration in the e-liquids tested (Fig 8A; S7 Data). Similarly, cinnamaldehyde toxicity was also proportional (R^2^ = 0.75) to the measured concentrations of cinnamaldehyde (Fig 8B; S7 Data). On the contrary, triacetin did not correlate to toxicity (R^2^ = 0.048; Fig 8C; S7 Data).

## Discussion

Fluorescent-based HTS techniques have been used for decades for drug discovery screens, toxicity, and for genetic screens, and their adoption by the scientific community has increased following the development of better fluorescent dyes and better instrumentation both in the pharmaceutical industry and academia [32, 33]. Despite the different endpoints, most HTS have common phases, including the initial phase (sometimes called the primary screen), which sets to capture as much preliminary data as possible—including false positives, which are subsequently confirmed or refuted. This key step allows for the inclusion of all possible prospective “hits” and minimization of lead loss. This is followed by the main (or secondary) screen in which extensive data are collected. Finally, validating (i.e., tertiary) screens are performed to confirm the main dataset. In our study, we performed the initial screen on 148 e-liquids to determine the optimum duration of the screen, find appropriate positive and negative controls, and to determine the most sensitive endpoint (Figs 1 and 2; S1 and S2 Data). Our colleagues have found that physiologic endpoints tend to be more sensitive than gross toxicological ones [34]. However, we chose assays that could be applied to most cells types, were readily available, and relatively easy to measure. We found that the viability assay, which meets these criteria, was more sensitive than the cell growth density (Fig 1F; S1 Data). That is, more e-liquids showed some alteration using the viability assay than the cell growth assay (126 e-liquids showed a decrease in viability, while 91 e-liquids showed a decrease in growth).

We used HEK293T cells for this screen because they are a cell line that is amenable to HTS and one of the favorites in the screening field [35, 36]. We also validated our findings using pulmonary cell lines. Importantly, we demonstrated that the toxicity seen in HEK293T cells was reproduced in hASMC and hA549 cells and the e-liquids showed similar rank order of toxicity in all cell lines (Fig 4; S4 Data). Similarly, using a 96-well–plate approach, we previously demonstrated that cultured human airway epithelial 3 (CALU3) cells showed differences in viability to 13 e-liquids [30]. In this study, we used the 3-(4,5-dimethylthiazol-2-yl)-2,5-diphenyl tetrazolium bromide (MTT) assay that measures dye uptake by live cells. However, we found that the MTT assay, while sensitive, was less amenable to HTS because it required more wash steps, which can cause cells to detach and required more preparation time. However, this assay may be useful as a validation step. It is unlikely that PG/VG will ever reach 10% or 30% in the lung lumen during normal vaping. However, we wanted to perform a full-dose response in order to fully understand the upper toxic limit of PG/VG. Accordingly, we can now say that it does not induce cell death below 1%. Furthermore, we have previously measured the P_O2_ in physiologic solutions (i.e., airway surface liquid) containing mucus gels that have exceeded 20% solids [1]. Despite a large amount of mucus and an extremely high viscous component, P_O2_ was normal, suggesting that similar levels of PG/VG will not alter P_O2_ levels. Other groups have reported cytotoxicity of e-liquids, but their screening capacity was 40 e-liquids or less [24, 37, 38]. Separately, studies have reported chemical composition analysis of e-liquids, but again, the total number was low [6, 39]. Overall, we are the first group to have designed and implemented a robust, high-throughput technique that allows for the parallel screening of hundreds of e-liquids.

Several researchers have found evidence of chemical transformation (i.e., pyrolysis) after vaping [5, 6]. However, in agreement with our previous study [30], we did not find that vaping e-liquids changed their relative toxicity, with the exception of one flavor (Hot Cinnamon Candy; Fig 5A and 5D; S5 Data), suggesting that this phenomenon may be flavor dependent. In our experience, vaping is more variable and less amenable to HTS. However, we vaped HEK293T cells, primary HBECs, and primary human macrophages. We found that the toxicity that occurred after vaping HEK293T cells and HBECs compared very well to the neat e-liquid addition (Fig 5D and 5F; S5 Data). Because the HBECs were well differentiated and exposed only apically under air–liquid interface conditions, this is perhaps the most realistic of conditions (Fig 5B and 5F; S5 Data). Similarly, after neat e-liquid exposure, hASM relative toxicities also correlated well with the HEK293T data (Fig 4; S4 Data). Surprisingly, the LC_50_ did not correlate at all with the vaping of macrophages (Fig 5E; S5 Data). Therefore, while we do not appear to observe a general increase in e-liquid toxicity after vaping as compared to liquid addition, the HTS data may not be representative of all pulmonary cell types. The actual deposition fraction of vaped e-liquids into the lungs remains to be determined. However, the predicted deposition of E-cig aerosol in the lungs is approximately 25% [40]. Therefore, if 1 ml of e-liquid is aerosolized and inhaled—and assuming a total airway surface liquid volume in the lung of approximately 3 ml—this would lead to 0.25 ml being deposited, suggesting a dilution factor of 1:12, or approximately 8%. Given that e-liquids have a LC_50_ of approximately 6% or less, this would suggest that e-liquids may reach biologically relevant levels in the lung. Indeed, it has recently been reported that vaping significantly alters the secreted human airway proteome, suggesting that this may be the case [41].

We are still understanding the relative toxicity of e-liquid constituents and their implications for airway exposure. PG is a common chemical used to produce polyester and as deicer/antifreeze, as well as being a base constituent in e-liquids. Intravenous PG can cause acute renal and central nervous system (CNS) toxicity [42], and PG inhalation causes renal and liver toxicity [43]. PG has previously been shown to inhibit renal glucose transport and corneal Na^+^/K^+^ATPase activity [44, 45]. Beyond PG, VG, and nicotine, we previously found no chemical similarity in 13 e-liquid flavors [30]. Therefore, given their heterogeneous nature, the overall goal of this project was to screen a greater number of neat e-liquids to identify flavors and/or chemical constituents that are more toxic and would direct additional studies. We found a number of highly toxic e-liquids that should be prioritized for study (Fig 1; S1 Table; S1 Data). Furthermore, in addition to identifying vanillin as potentially highly toxic, we also identified—using this screen—3 e-liquids that contain diacetyl (2,3-butanedione), which causes bronchiolitis obliterans, and 5 e-liquids that contain 2,3-butanedione monooxime, which is a chemical diphosphatase that blocks ATP-sensitive K^+^ channels [46–51]. Interestingly, many flavors, e.g., benzaldehyde (almond) and cinnamaldehyde (cinnamon), are aldehydes that can form protein adducts [52]. Benzaldehyde was only detected in 4 e-liquids. Cinnamaldehyde was found in 8 e-liquids (including Cinnamon Roll, Hot Cinnamon Candies, and Root Beer) and has previously been shown to impair phagocytosis in macrophages [34]. Sherwood and Boitano [24] recently exposed airway epithelia to 7 chemical flavors and concluded that vanillin and a chocolate flavor (2,5-dimethypyrazine) had the biggest effect on their cells. We found that vanillin was present in 63 out of 148 e-liquids. However, 2,5-dimethypyrazine was not detected in any of our e-liquids. Vanillin activates transient receptor potential cation channel subfamily V member (TRPV) channels. TRPV channels are expressed in neurons and serve as nonselective cation channels that can increase cytoplasmic Ca^2+^ levels in epithelia [53]. Of note, prolonged increases in cytosolic Ca^2+^ can alter cell division rates and are indicative of apoptosis [54].

Using an NMDS approach, we found only a weak correlation between the presence of flavorings and toxicity (i.e., LC_50_). Of the e-liquids shown in Fig 7A and 7B (S6 Data), all had a constant ratio of 55% PG to 45% VG, indicating that PG/VG did not influence the change in toxicity. Furthermore, most flavors analyzed in Fig 7A (S6 Data) had 12 mg/ml nicotine, suggesting that changes in nicotine levels also did not account for the variability. However, of the 148 e-liquids tested, we found approximately 123 different chemicals. Therefore, we may have to further expand our HTS dataset in order to obtain a better correlation between the presence/absence of e-liquid constituents and toxicity, assuming that if we expand the dataset from 148 to 500 or 1,000 e-liquids, the number of chemical constituents eventually levels off and does not increase proportionally. We also determined the concentrations of cinnamaldehyde, vanillin, and triacetin against known concentrations of these compounds (see Fig 6C). Using this approach, we found a positive correlation between vanillin (0.62) and cinnamaldehyde (0.75) but not triacetin (0.048), with concentration/toxicity (Fig 8; S7 Data), suggesting that other chemicals present in these e-liquids may also have influence. Therefore, while we now have a better appreciation of the range of toxicities of different e-liquids, further work will be required to fully understand which additional components influence toxicity. A limitation of the proposed studies is that the chemical of interest will need to be available for purchase or be synthesizable and purifiable in order to be quantified by GC-MS or similar techniques.

To further categorize and evaluate these data, we have developed a database (www.eliquidinfo.org). This website is publicly available, and in its current form, it is most likely to be useful to academic and government researchers. Through this portal, one can browse LC_50_ values, search for different chemicals, and determine which e-liquids contain them. Given the diversity of e-liquid toxicity and composition, we have found this website extremely useful in choosing new e-liquids for future studies. For example, we now select some less toxic, intermediate, and more toxic e-liquids when starting new investigations rather than just study one e-liquid. As we complete additional rounds of HTS, we hope to grow this database to make it more applicable. However, this website may also serve to inform the general public as to the relative toxicity and heterogeneity of e-liquids.

Given that the rise in vaping popularity has vastly outstripped our knowledge of its potential health benefits versus potential adverse effects, such HTS approaches will allow us to rapidly screen the approximately 7,700 different e-liquids that are on the market [10]. Whether or not PG/VG and nicotine are less harmful than inhaled tobacco is highly contentious [55]. However, HTS approaches for both e-liquids and their chemical constituents still have an important role in helping to shape future legislation for e-liquids and vaping. This is becoming all the more important, especially as researchers from the tobacco industry are now making claims that vaping represents a reduced risk of exposure compared to tobacco smoking [55, 56]. Therefore, it is vital that academic and government laboratories independently test as many different classes of these e-liquids as possible using multiple approaches and use evidence-based research as the guide for regulation. For example, when low-tar cigarettes were introduced and producers claimed they were a safer alternative to traditional cigarettes, these claims were later refuted. Given the claims of some groups, including tobacco companies and the public perception that e-liquids are safer than tobacco products, such an approach to study e-liquid toxicity may serve as a roadmap to enable bodies such as the US FDA to properly regulate e-liquid manufacturing and sale.

## Methods

### Flavored e-liquids

Flavored e-liquids were purchased from The Vapor Girl (www.thevaporgirl.com), NJOY (https://www.njoy.com), and E-TONIC (https://www.hookah-shisha.com). The e-liquids contained a variety of nicotine concentrations, ranging from 0 to 12 mg/mL, and a PG to VG ratio of 55:45. Therefore, a 55/45 PG/VG vehicle control was made in our laboratory using chemicals purchased from Sigma-Aldrich (St. Louis, MO). For more information about the e-liquids, see S1 Table.

### Chemicals and reagents

PG (FG grade), VG (FG grade), and DMSO (ACS grade) were purchased from Sigma-Aldrich. Calcein-AM and propidium iodide were purchased from Thermo-Fisher (Waltham, MA). A modified Ringers solution (101 mM NaCl, 12 mM NaHCO_3_, 24 mM HEPES, 1.2 mM MgCl_2_, 1.2 mM CaCl_2_∙2 H_2_O, 5.2 mM KCl, and 10 mM D-(+)-Glucose) was made with chemicals purchased from Sigma-Aldrich (all ACS grade). Cell culture reagents were obtained from Gibco (Waltham, MA).

### Cell culture

HEK239T cells were incubated at 37 °C with 5% CO_2_ and cultured in DMEM supplemented with 10% FBS and 1X penicillin/streptomycin. hA549 cells were incubated at 37 °C with 5% CO_2_ and cultured in RMPI 1640 supplemented with 10% FBS and 1X penicillin/streptomycin.

Primary HBECs and hASMCs were harvested by enzymatic digestion of human bronchial tissue obtained from donor lungs using protocols approved by the University of North Carolina at Chapel Hill Committee on the Protection of the Rights of Human Subjects. HBECs were plated on 6.5 mm Transwell T-col culture inserts (Coning, NY) and cultured at the air–liquid interface in UNC air–liquid interface media for 28 days before use as previously described [57]. hASMCs were cultured in 384-well plates, incubated at 37 °C with 5% CO_2_, and cultured in DMEM-α supplemented with 10% FBS and 1X penicillin/streptomycin using passages 3–6 [29].

Bronchoalveolar lavage fluid was obtained from healthy human subjects under a protocol approved by the University of North Carolina at Chapel Hill Committee on the Protection of the Rights of Human Subjects (#91–0679). All patients included in this study gave their written informed consent. Airway macrophage (AM) isolation was performed as previously described [58]. In brief, the cell pellet was resuspended in macrophage medium (RPMI 1640, 10% FBS, 100 U/ml penicillin, 100 μg/ml streptomycin). Following a 3-h adherence at 37 °C, 5% CO_2_, supernatants were removed, and adherent cells were washed 5 times with PBS. Cell preparations typically consisted of >98% AMs. Freshly isolated AMs were seeded onto 96-well plates at a concentration of 10,000 AMs per well and cultured in macrophage medium for the duration of the experiment.

### Primary viability screen

HEK293T cells were plated on poly-L-lysine–coated 384-well plates from Corning (Corning, NY) at a density of 5,000 cells per well at t = 0 and incubated at 37 °C, 5% CO_2_ for 4 h to allow cells to adhere. Cells were imaged with a Cytation5 imaging plate reader (BioTek, Winooski, VT) using the bright-field feature to establish baseline surface area. After 4 h, cells were treated with e-liquids at a concentration of 1% (*n =* 4) and returned to the Cytation5, and images were acquired every 2 h for 24 h. Controls included PBS (negative), vehicle (10% 55:45 PG/VG, positive), and media (baseline). At t = 30–32 h, media were replaced with a modified Ringers solution containing calcein-AM (3 μM) and propidium iodide (3 μM) and incubated for 30 min to measure cell viability. The ratio of the fluorescence intensity of calcein and propidium iodide was normalized to media controls. Gen5 2.09 software (Biotek) was used to acquire bright-field images, and ImageJ (NIMH, Bethesda, MD) was used to calculate covered surface area to assess cell growth.

### Secondary viability screen

HEK293T cells were plated on poly-L-lysine–coated 384-well plates (Corning, NY) at a density of 5,000 cells per well at t = 0 and incubated for 4 h to allow cells to adhere. At that time, cells were treated with various concentrations of e-liquid diluted in media for 24 h, including PBS (negative), vehicle (PG/VG, positive), and media controls. At t = 28–30 h, media were replaced with a modified Ringers solution containing calcein-AM (3 μM) and propidium iodide (3 μM) as a live/dead cell stain and incubated for 30 min. Cells were then imaged with a Cytation5 imaging plate reader (BioTek). The ratio of the fluorescence intensity of calcein and propidium iodide was normalized to media controls and plotted as 8- or 16-point dose–response curves. A nonlinear 4-parameter regression was conducted, and the LC_50_ value was determined for each e-liquid using GraphPad Prism6 (La Jolla, CA).

### Cell line validation assay

Cells were plated on poly-L-lysine–coated 384-well plates at a density of 5,000 cells per well for A549 and 1,000 cells per well for hASMC cells at t = 0 and incubated for 4 to 6 h to allow cells to adhere. At that time, cells were treated with various concentrations of e-liquid diluted in media for 22 to 24 h, including PBS (negative), vehicle (PG/VG, positive), and media controls. At t = 28–30 h, media were replaced with a modified Ringers solution containing calcein-AM (3 μM) and propidium iodide (3 μM) as a live/dead cell stain and incubated for 30 min. Cells were then imaged with a Cytation5 imaging plate reader (BioTek). The ratio of the fluorescence intensity of calcein and propidium iodide was normalized to media controls and plotted as 8- or 16-point dose–response curves; each dose was run in triplicate (*n =* 3) on 3 independent occasions (*N =* 3). A nonlinear 4-parameter regression was conducted, and the LC_50_ value was determined for each e-liquid (GraphPad Prism6).

### Manual E-cig and aerosol generation

E-cig aerosols were generated using a Sigelei FuChai 200 W device with a Crown stainless steel subtank and a 0.25 Ω SUS316 dual coil from Uwell (City of Industry, CA). Aerosols were generated by activating the E-cig device and drawing into a 100 mL syringe from the mouthpiece of the subtank. Based on existing E-cig topography [59–61], we generated 70 mL puffs drawn over 4 s and dispensed with a flow rate of 0.84 L/min at 100 W, unless otherwise stated. To directly vape into 96-well plates, we used a 3D printed manifold as previously described [31]. These manifolds were used to simultaneously vape 6 wells per plate. Cells were exposed to 10 puffs of vaped e-liquid as indicated above. We have shown that e-liquids are autofluorescent, and using autofluorescence as an indicator of deposition, we previously found that our vaping approach in 96-well plates resulted in an even deposition of e-liquid vapor that was highly reproducible [31].

### Automated E-cig vapor exposure of HBECs

HBECs were incubated apically for 20 min with PBS to remove excess mucus 24 h before exposure. On the day of exposure, cultures were loaded into the exposure block of a VC10 smoking robot (Vitrocell, Germany) with each culture insert exposed to E-cig vapor from a Sigelei Fuchai 200 W third-generation device set to 100 W, using Uwell Crown tanks with 0.25 Ω dual coils. The device was activated by a pneumatic actuator integrated into the system and connected directly to the syringe pump of the VC10 before a triangle curve puff was applied over 4 s for a volume of 70 mL and exhausted over 8 s with a period of 30 s. The vapor flowed into the 24 wells of the exposure block, with each well being fed directly by a “trumpet” allowing the vapor access to each HBEC mucosal surface. Serosally, the inserts were in contact with ALI media and were maintained at 37 °C throughout the exposure period. Cells were exposed to 70 puffs using the puff parameters described above. After the exposure, the cells were replaced in 24-well plates and returned to the 37 °C, 5% CO_2_ incubator for 24 h. The lines of the VC10 were also exposed to filtered compressed air to flush the majority of the vapor condensate from the lines, pump cylinder, and exposure apparatus before the next exposure with a new e-liquid, and the entire system was cleaned after each vaping session.

### Aerosol validation assay

HEK293T cells were plated on poly-L-lysine–coated 96-well plates (Corning, NY) at a density of 30,000 cells per well at t = 0 and incubated for 4 to 6 h to allow cells to adhere. AMs were plated as described above. Cells were exposed to 10 puffs of vaped e-liquids using a 4 s, 70 ml puff and incubated for 22 to 24 h. Media were then replaced with a modified Ringers solution containing calcein-AM (3 μM) and propidium iodide (3 μM) and incubated for 30 min to measure viability stain. Cells were then imaged using a Cytation5 imaging plate reader (BioTek). The normalized ratio of the average fluorescence intensity of calcein and propidium iodide was reported.

### O_2_ measurements

The P_O2_ was measured using a modification of our previous method [62]. In brief, the voltage output from a solid-state O_2_ electrode (STDO11) from Ohaus (Parsippany, NJ) was read using a pH/voltage meter (Thermo-Fisher, Waltham, MA) operating in voltage mode. The O_2_ electrode was calibrated using media with atmospheric O_2_ (i.e., approximately 21% O_2_), and media bubbled for 2 h with 100% N_2_ (i.e., 0% O_2_). Cell culture media ± 30% PG/VG were added to HEK293T cells for 24 h, and O_2_ levels were read immediately after calibration.

### GC-MS analysis of e-liquids

Qualitative e-liquid analysis was performed on a Bruker EVOQ 456 gas chromatograph-triple quadrupole mass spectrometer (Billerica, MA) using an Agilent DB-5MS capillary column (30 m, 0.25 mm ID, 0.25 μM film) and helium carrier gas (99.999% purity; Santa Clara, CA). Injections (1 μL) were performed using a Bruker CP-8400 autosampler with an injector temperature of 270 °C. The GC oven was programmed with a 12.5 min temperature gradient (60–250 °C), and the transfer line and EI source were held at 250 °C. Samples were prepared by diluting 50 μL of e-liquid in 1 mL of methanol (optima grade) and vortexing for 30 s. Full-scan mass spectra were acquired from *m/z* 40–500. Compound identification was performed using the NIST 2014 mass spectral database (Gaithersburg, MD) and AMDIS chromatography software.

For the quantitative process, flavor concentrations were determined by standard addition. E-liquids were diluted in methanol (optima grade) and quantitative standards. A full list of e-liquid dilutions and standard concentrations is given in S2 Table. Selected ion monitoring (SIM) mass spectra were acquired for each of the quantified flavors. SIM parameters are given in S3 Table. Peak areas of quantitative ions were integrated for quantification of each of the flavors. Qualitative ions were used for confirmation of compound identity.

### Statistics and bioinformatics analysis of e-liquid population

All experiments were performed on a minimum of 3 separate occasions (*N =* 3). All data are shown as mean ± standard error, such that “n” refers to the number of plates or donors as appropriate. For 384-well–plate experiments, each dose was performed in triplicate per plate. All statistic and curve plotting were performed using Prism 6 (GraphPad, La Jolla, CA).

An ordination technique, NMDS was applied in R version 3.3.3 [63] using the package “vegan” [64] to matrices containing e-liquids and their binary (presence/absence) chemical composition. The same data table was clustered using *k-*modes (k = 2) using the package “klaR” [65], and chemicals within each cluster were compared using a Welch two-sample *t* test, in which resultant *p*-values were adjusted using Bonferroni correction.

## Supporting information

S1 Table E-liquid properties and the LC_50_ values obtained from the viability (calcein/propidium iodide) assay. Mean data were obtained in HEK293T cell cultures in triplicate in 384-well plates on 3 separate occasions. For images of all dose–response curves, please visit www.eliquidinfo.org. Raw data are available in S8 Data. HEK293T, human embryonic kidney 293 cells; IRN, internal reference number; LC_50_, concentration at which a given agent is lethal to 50% of the cells; SEM, standard error of the mean.
(DOCX)

S2 Table Sample preparation for e-liquid quantification. (DOCX)

S3 Table SIM parameters used for quantification of e-liquid flavors. SIM, selected ion monitoring.
(DOCX)

S1 Data Fig 1: Development of preliminary screens to assess e-liquid toxicity in vitro. (XLSX)

S2 Data Fig 2: PG/VG alone negatively effects cell viability. PG, propylene glycol; VG, vegetable glycerin.
(XLSX)

S3 Data Fig 3: Main screen used to assess e-liquid toxicity. (XLSX)

S4 Data Fig 4: Orthogonal assays to validate human airway cell types. (XLSX)

S5 Data Fig 5: Toxicity of “vaped” versus neat e-liquids. (XLSX)

S6 Data Fig 7: The presence/absence of e-liquid constituents and their toxicity have some correlation. (XLSX)

S7 Data Fig 8: Vanillin and cinnamaldehyde concentrations correlate with toxicity in select e-liquids. (XLSX)

S8 Data S1 Table: E-liquid properties and the LC50 values obtained from the viability (calcein/propidium iodide) assay. (XLSX)

## Abbreviations

AM: airway macrophage
CALU3: cultured human airway epithelial 3 cells
CNS: central nervous system
DMSO: dimethyl sulfoxide
E-cig: electronic cigarette
ENDS: electronic nicotine delivery system
FDA: Food and Drug Administration
GC-MS: gas chromatography–mass spectrometry
GRAS: Generally Recognized As Safe
hA549: human adenocarcinomic alveolar basal epithelial cells
hASMC: human airway smooth muscle cell
HBEC: human bronchial epithelial cells
HEK293T: human embryonic kidney 293 cells
HTS: high-throughput screening
LC_50_: concentration at which a given agent is lethal to 50% of the cells
MTT assay: (4,5-dimethylthiazol-2-yl)-2,5-diphenyl tetrazolium bromide assay
NIST: National Institute of Standards and Technology
NMDS: nonmetric multidimensional scaling
PBS: phosphate-buffered saline
PG: propylene glycol
P_O2_: partial pressure of O_2_
SIM: selected ion monitoring
TRPV: transient receptor potential cation channel subfamily V member
VG: vegetable glycerin
