# Supplementary material for: Evaluation of e-liquid toxicity using an open-source high-throughput screening assay
Source: PLoS Biol. 2018 Mar 27;16(3):e2003904. doi: 10.1371/journal.pbio.2003904 (PMC5870948; doi:10.1371/journal.pbio.2003904)
Supplement: S1 Table — Mean data were obtained in HEK293T cell cultures in triplicate in 384-well plates on 3 separate occasions. For images of all dose–response curves, please visit www.eliquidinfo.org. Raw data are available in S8 Data. HEK293T, human embryonic kidney 293 cells; IRN, internal reference number; LC50, concentration at which a given agent is lethal to 50% of the cells; SEM, standard error of the mean. (DOCX) [file pbio.2003904.s001.docx]

**S1 Table.** E-liquid properties, and the LC_50_ values obtained from the viability (calcein/propidium iodide) assay. Mean data were obtained in HEK293T cells cultures in triplicate in 384 well plates on 3 separate occasions. IRN: Internal reference number, LC50: concentration of a given agent that is lethal to 50% of the cells, SEM: standard error of the mean. For images of all dose-response curves, please visit [www.eliquidinfo.org](http://www.eliquidinfo.org).

| **Internal reference number (IRN)** | **E-liquid Name** | **LC_50_ (% v/v)** | | **Log[LC_50_] ± SEM** | **Bottom ± SEM** | **Top ± SEM** | **Span ± SEM** | **Hill Slope ± SEM** | | **Lot Number** | **Nicotine (mg/mL)** | | **PG/VG Ratio** | **Vendor** |
| --- | --- | --- | --- | --- | --- | --- | --- | --- | --- | --- | --- | --- | --- | --- |
| 69 | 1 Leaf Tobacco | | 3.66 | 0.56 ± 0.06 | 0.04 ± 0.07 | 0.92 ± 0.02 | 0.88 ± 0.08 | -1.4 ± 0.3 | 114414 | | | 12 | 55/45 | The Vapor Girl Inc. |
| 65 | 10 Leaves Tobacco | | 4.50 | 0.65 ± 0.03 | 0.42 ± 0.03 | 1.04 ± 0.02 | 0.62 ± 0.03 | -3.0 ± 0.5 | | 114414 | 12 | | 55/45 | The Vapor Girl Inc. |
| 61 | 5 Leaves Tobacco | | 3.10 | 0.49 ± 0.04 | 0.30 ± 0.04 | 1.06 ± 0.02 | 0.76 ± 0.04 | -1.8 ± 0.4 | | 114414 | 12 | | 55/45 | The Vapor Girl Inc. |
| 34 | Alchemy | | 2.93 | 0.468 ± 0.006 | 0.29 ± 0.01 | 1.12 ± 0.01 | 0.83 ± 0.02 | -9.1 ± 1.6 | | 114414 | 12 | | 55/45 | The Vapor Girl Inc. |
| 27 | Ambrosia | | 2.84 | 0.45 ± 0.01 | 0.29 ± 0.01 | 1.08 ± 0.01 | 0.80 ± 0.02 | -3.7 ± 0.6 | | 114414 | 12 | | 55/45 | The Vapor Girl Inc. |
| 23 | Angel Lust | | 1.54 | 0.19 ± 0.01 | 0.155 ± 0.009 | 1.054 ± 0.006 | 0.90 ± 0.01 | -2.6 ± 0.1 | | 114414 | 12 | | 55/45 | The Vapor Girl Inc. |
| 122 | Apple Pie | | 1.29 | 0.11 ± 0.02 | 0.12 ± 0.01 | 1.01 ± 0.01 | 0.88 ± 0.02 | -1.7 ± 0.1 | | 114414 | 12 | | 55/45 | The Vapor Girl Inc. |
| 62 | Arctic Blueberry | | 4.58 | 0.66 ± 0.06 | 0.11 ± 0.05 | 1.07 ± 0.01 | 0.96 ± 0.06 | -2.4 ± 0.5 | | 114414 | 12 | | 55/45 | The Vapor Girl Inc. |
| 78 | Arctic Raspberry | | 1.36 | 0.13 ± 0.04 | 0.15 ± 0.02 | 1.00 ± 0.02 | 0.85 ± 0.03 | -2.4 ± 0.4 | | 114414 | 12 | | 55/45 | The Vapor Girl Inc. |
| 102 | Arctic Strawberry | | 4.05 | 0.61 ± 0.04 | 0.18 ± 0.04 | 1.090 ± 0.009 | 0.91 ± 0.04 | -2.4 ± 0.4 | | 114414 | 12 | | 55/45 | The Vapor Girl Inc. |
| 68 | Arctic Tobacco | | 0.30 | -0.52 ± 0.07 | 0.22 ± 0.03 | 1.07 ± 0.01 | 0.86 ± 0.04 | -1.1 ± 0.2 | | 114414 | 12 | | 55/45 | The Vapor Girl Inc. |
| 158 | Bada Bing! | | 5.67 | 0.75 ± 0.04 | 0.42 ± 0.03 | 0.90 ± 0.02 | 0.48 ± 0.04 | -4.7 ± 1.7 | | L1 | 0 | | 55/45 | E-Tonic |
| 45 | Bahama Mama | | 4.99 | 0.70 ± 0.02 | 0.14 ± 0.03 | 1.05 ± 0.01 | 0.90 ± 0.03 | -2.0 ± 0.2 | | 114414 | 12 | | 55/45 | The Vapor Girl Inc. |
| 114 | Banana | | 1.48 | 0.17 ± 0.03 | 0.14 ± 0.02 | 1.14 ± 0.01 | 1.00 ± 0.02 | -2.3 ± 0.2 | | 114414 | 12 | | 55/45 | The Vapor Girl Inc. |
| 42 | Banana Nut Bread Smoothie | | 1.75 | 0.24 ± 0.03 | 0.16 ± 0.01 | 1.01 ± 0.01 | 0.85 ± 0.02 | -3.7 ± 0.5 | | 114414 | 12 | | 55/45 | The Vapor Girl Inc. |
| 320 | Banana Nut  Bread Smoothie 2 | | 1.38 | 0.14 ±  0.04 | 0.15 ±  0.03 | 1.01 ±  0.01 | 0.86 ±  0.03 | -2.1 ±  0.0.4 | | 111816 | 12 | | 55/45 | The Vapor Girl Inc |
| 166 | Banana Pudding | | 0.79 | -0.10 ± 0.05 | 0.13 ± 0.02 | 1.13 ± 0.02 | 1.00 ± 0.03 | -3.8 ± 1.7 | | 114414 | 0 | | 55/45 | The Vapor Girl Inc. |
| 129 | Banana Pudding Southern Style | | 0.67 | -0.17 ± 0.04 | 0.15 ± 0.03 | 1.12 ± 0.02 | 0.98 ± 0.03 | -2.7 ± 0.5 | | 114414 | 12 | | 55/45 | The Vapor Girl Inc. |
| 165 | Banana Pudding Southern Style | | 1.16 | 0.06 ± 0.03 | 0.18 ± 0.02 | 1.09 ± 0.02 | 0.91 ± 0.03 | -2.2 ± 0.3 | | 114909 | 0 | | 55/45 | The Vapor Girl Inc. |
| 63 | Banana Yo! (Yogurt!) | | 3.56 | 0.55 ± 0.02 | 0.31 ± 0.02 | 1.07 ± 0.02 | 0.75 ± 0.03 | -3.4 ± 0.5 | | 114414 | 12 | | 55/45 | The Vapor Girl Inc. |
| 49 | Barlett Pear | | 3.23 | 0.51 ± 0.01 | 0.33 ± 0.02 | 1.04 ± 0.01 | 0.71 ± 0.02 | -6.0 ± 1.2 | | 114414 | 12 | | 55/45 | The Vapor Girl Inc. |
| 26 | Battle Frogs | | 3.20 | 0.51 ± 0.02 | 0.39 ± 0.02 | 1.08 ± 0.02 | 0.70 ± 0.02 | -4.0 ± 0.8 | | 114414 | 12 | | 55/45 | The Vapor Girl Inc. |
| 115 | Bavarian Creme Donut | | 0.59 | -0.23 ± 0.03 | 0.139 ± 0.009 | 0.98 ± 0.01 | 0.84 ± 0.02 | -1.5 ± 0.1 | | 114414 | 12 | | 55/45 | The Vapor Girl Inc. |
| 89 | Biscotti | | 0.69 | -0.16 ± 0.04 | 0.07 ± 0.02 | 1.08 ± 0.02 | 1.01 ± 0.03 | -2.2 ± 0.3 | | 114414 | 12 | | 55/45 | The Vapor Girl Inc. |
| 142 | Black and Blue Berries | | 2.45 | 0.39 ± 0.02 | 0.29 ± 0.01 | 1.02 ± 0.01 | 0.72 ± 0.02 | -3.2 ± 0.6 | | Unknown | 10 | | 55/45 | NJOY |
| 125 | Black Cherry | | 3.40 | 0.53 ± 0.04 | 0.09 ± 0.05 | 1.13 ± 0.01 | 1.03 ± 0.05 | -2.5 ± 0.7 | | 114414 | 12 | | 55/45 | The Vapor Girl Inc. |
| 90 | Black Coffee | | 0.63 | -0.20 ± 0.05 | 0.18 ± 0.02 | 1.09 ± 0.01 | 0.92 ± 0.02 | -3.8 ± 0.8 | | 114414 | 12 | | 55/45 | The Vapor Girl Inc. |
| 20 | Black Dragon | | 4.40 | 0.64 ± 0.02 | 0.31 ± 0.02 | 1.058 ± 0.008 | 0.75 ± 0.02 | -3.0 ± 0.3 | | 114414 | 12 | | 55/45 | The Vapor Girl Inc. |
| 80 | Black Hawk | | 3.11 | 0.49 ± 0.01 | 0.23 ± 0.02 | 1.09 ± 0.02 | 0.86 ± 0.03 | -5.5 ± 1.4 | | 114414 | 12 | | 55/45 | The Vapor Girl Inc. |
| 57 | Black Honey Tobacco | | 3.13 | 0.50 ± 0.02 | 0.21 ± 0.02 | 1.07 ± 0.01 | 0.86 ± 0.03 | -3.0 ± 0.5 | | 114414 | 12 | | 55/45 | The Vapor Girl Inc. |
| 12 | Black Licorice | | 3.75 | 0.57 ± 0.02 | 0.18 ± 0.02 | 0.99 ± 0.01 | 0.81 ± 0.02 | -2.7 ± 0.3 | | 114414 | 12 | | 55/45 | The Vapor Girl Inc. |
| 101 | Black Peppercorn | | 2.75 | 0.44 ± 0.03 | 0.41 ± 0.02 | 1.04 ± 0.02 | 0.62 ± 0.03 | -5.1 ± 2.4 | | 114414 | 12 | | 55/45 | The Vapor Girl Inc. |
| 109 | Black Thorn | | 4.08 | 0.61 ± 0.02 | 0.27 ± 0.02 | 1.032 ± 0.008 | 0.77 ± 0.02 | -2.9 ± 0.3 | | 114414 | 12 | | 55/45 | The Vapor Girl Inc. |
| 53 | Blackberry Lemonade | | 3.05 | 0.48 ± 0.01 | 0.30 ± 0.02 | 1.07 ± 0.02 | 0.78 ± 0.02 | -6.0 ± 1.4 | | 114414 | 12 | | 55/45 | The Vapor Girl Inc. |
| 44 | Blackberry Panda | | 3.10 | 0.491 ± 0.007 | 0.28 ± 0.01 | 1.07 ± 0.01 | 0.79 ± 0.02 | -7.8 ± 1.3 | | 114414 | 12 | | 55/45 | The Vapor Girl Inc. |
| 141 | Blood Orange | | 2.23 | 0.35 ± 0.05 | 0.10 ± 0.04 | 1.10 ± 0.03 | 1.00 ± 0.05 | -2.0 ± 0.4 | | A4F32 | 10 | | 55/45 | NJOY |
| 59 | Blue DUDE | | 3.59 | 0.56 ± 0.02 | 0.45 ± 0.02 | 1.07 ± 0.02 | 0.62 ± 0.03 | -4.1 ± 0.9 | | 114414 | 12 | | 55/45 | The Vapor Girl Inc. |
| 106 | Blue Moo | | 1.84 | 0.27 ± 0.02 | 0.14 ± 0.01 | 1.09 ± 0.01 | 0.94 ± 0.02 | -3.7 ± 0.4 | | 114414 | 12 | | 55/45 | The Vapor Girl Inc. |
| 76 | Blue Pom (Pomegranate) | | 4.75 | 0.68 ± 0.04 | 0.18 ± 0.04 | 1.109 ± 0.008 | 0.93 ± 0.04 | -2.0 ± 0.3 | | 114414 | 12 | | 55/45 | The Vapor Girl Inc. |
| 100 | Blueberry Cinnamon-Streusal Muffin | | 1.63 | 0.21 ± 0.02 | 0.22 ± 0.01 | 1.066 ± 0.008 | 0.84 ± 0.02 | -1.7 ± 0.1 | | 114414 | 12 | | 55/45 | The Vapor Girl Inc. |
| 55 | Blueberry Lemonade | | 3.80 | 0.58 ± 0.02 | 0.35 ± 0.02 | 1.03 ± 0.01 | 0.68 ± 0.02 | -3.6 ± 0.5 | | 114414 | 12 | | 55/45 | The Vapor Girl Inc. |
| 39 | Blueberry Tobacco | | 6.00 | 0.78 ± 0.02 | 0.21 ± 0.02 | 1.05 ± 0.01 | 0.84 ± 0.03 | -2.3 ± 0.2 | | 114414 | 12 | | 55/45 | The Vapor Girl Inc. |
| 67 | Briar Patch | | 3.91 | 0.59 ± 0.02 | 0.23 ± 0.02 | 1.05 ± 0.01 | 0.82 ± 0.02 | -2.7 ± 0.3 | | 114414 | 12 | | 55/45 | The Vapor Girl Inc. |
| 50 | Brittany's Blue Blend | | 3.67 | 0.56 ± 0.02 | 0.35 ± 0.02 | 1.00 ± 0.01 | 0.65 ± 0.02 | -5.4 ± 1.0 | | 114414 | 12 | | 55/45 | The Vapor Girl Inc. |
| 56 | Bubble Gum | | 3.68 | 0.57 ± 0.02 | 0.33 ± 0.02 | 1.03 ± 0.02 | 0.70 ± 0.02 | -3.9 ± 0.6 | | 114414 | 12 | | 55/45 | The Vapor Girl Inc. |
| 79 | Bubbly Berry | | 3.24 | 0.51 ± 0.02 | 0.20 ± 0.03 | 1.05 ± 0.02 | 0.85 ± 0.04 | -4.0 ± 1.0 | | 114414 | 12 | | 55/45 | The Vapor Girl Inc. |
| 135 | Butter Crunch | | 2.61 | 0.42 ± 0.05 | 0.08 ± 0.05 | 1.15 ± 0.01 | 1.08 ± 0.05 | -1.6 ± 0.3 | | A4727 | 10 | | 55/45 | NJOY |
| 112 | Butterscotch | | 2.14 | 0.33 ± 0.02 | 0.12 ± 0.02 | 1.10 ± 0.01 | 0.98 ± 0.02 | -2.4 ± 0.3 | | 114414 | 12 | | 55/45 | The Vapor Girl Inc. |
| 96 | Buttery Nipple | | 3.04 | 0.48 ± 0.02 | 0.13 ± 0.02 | 1.01 ± 0.02 | 0.88 ± 0.03 | -4.4 ± 1.1 | | 114414 | 12 | | 55/45 | The Vapor Girl Inc. |
| 60 | Candy Cane | | 0.89 | -0.05 ± 0.03 | 0.14 ± 0.02 | 1.09 ± 0.01 | 0.94 ± 0.02 | -2.9 ± 0.7 | | 114414 | 12 | | 55/45 | The Vapor Girl Inc. |
| 43 | Candy Corn | | 2.26 | 0.35 ± 0.01 | 0.103 ± 0.009 | 1.031 ± 0.006 | 0.93 ± 0.01 | -2.3 ± 0.1 | | 114414 | 12 | | 55/45 | The Vapor Girl Inc. |
| 19 | Captain Suckle | | 3.25 | 0.51 ± 0.01 | 0.29 ± 0.02 | 1.12 ± 0.01 | 0.83 ± 0.02 | -3.7 ± 0.5 | | 114414 | 12 | | 55/45 | The Vapor Girl Inc. |
| 47 | Captain Zack | | 3.71 | 0.57 ± 0.01 | 0.19 ± 0.02 | 1.056 ± 0.009 | 0.87 ± 0.02 | -3.2 ± 0.3 | | 114414 | 12 | | 55/45 | The Vapor Girl Inc. |
| 128 | Captain Zack Cigar | | 4.33 | 0.64 ± 0.04 | 0.16 ± 0.04 | 1.11 ± 0.01 | 0.95 ± 0.04 | -2.6 ± 0.5 | | 1523673 | 0 | | 55/45 | The Vapor Girl Inc. |
| 111 | Caramel Apple | | 1.24 | 0.09 ± 0.02 | 0.13 ± 0.02 | 1.13 ± 0.01 | 1.01 ± 0.02 | -2.8 ± 0.4 | | 114414 | 12 | | 55/45 | The Vapor Girl Inc. |
| 64 | Caramel Corn Crunch | | 2.93 | 0.47 ± 0.02 | 0.22 ± 0.02 | 1.04 ± 0.01 | 0.82 ± 0.02 | -2.6 ± 0.4 | | 114414 | 12 | | 55/45 | The Vapor Girl Inc. |
| 105 | Cat Nip | | 2.16 | 0.33 ± 0.02 | 0.20 ± 0.01 | 1.101 ± 0.009 | 0.90 ± 0.01 | -3.4 ± 0.3 | | 114414 | 12 | | 55/45 | The Vapor Girl Inc. |
| 84 | Chai | | 3.33 | 0.52 ± 0.04 | 0.05 ± 0.04 | 1.06 ± 0.01 | 1.01 ± 0.04 | -1.9 ± 0.3 | | 114414 | 12 | | 55/45 | The Vapor Girl Inc. |
| 85 | Chai Latte | | 2.72 | 0.43 ± 0.02 | 0.12 ± 0.02 | 1.06 ± 0.02 | 0.94 ± 0.03 | -4.3 ± 1.2 | | 114414 | 12 | | 55/45 | The Vapor Girl Inc. |
| 97 | Cheesecake with Graham Cracker Crust | | 2.93 | 0.47 ± 0.02 | 0.22 ± 0.02 | 1.06 ± 0.01 | 0.84 ± 0.02 | -2.7 ± 0.4 | | 114414 | 12 | | 55/45 | The Vapor Girl Inc. |
| 16 | Cherry Kola | | 0.48 | -0.31 ± 0.04 | 0.09 ± 0.02 | 0.99 ± 0.01 | 0.90 ± 0.02 | -2.1 ± 0.3 | | 114414 | 12 | | 55/45 | The Vapor Girl Inc. |
| 37 | Chicken and Waffles | | 5.37 | 0.73 ± 0.02 | 0.19 ± 0.02 | 1.06 ± 0.01 | 0.87 ± 0.03 | -2.2 ± 0.2 | | 114414 | 12 | | 55/45 | The Vapor Girl Inc. |
| 87 | Chocolate Banana | | 0.41 | -0.39 ± 0.03 | 0.09 ± 0.01 | 1.12 ± 0.01 | 1.03 ± 0.02 | -2.3 ± 0.3 | | 114414 | 12 | | 55/45 | The Vapor Girl Inc. |
| 54 | Chocolate Covered Raisins | | 0.76 | -0.12 ± 0.02 | 0.214 ± 0.007 | 1.037 ± 0.007 | 0.82 ± 0.01 | -3.1 ± 0.4 | | 114414 | 12 | | 55/45 | The Vapor Girl Inc. |
| 104 | Chocolate Dipt Raspberries | | 1.86 | 0.27 ± 0.02 | 0.26 ± 0.01 | 1.07 ± 0.01 | 0.81 ± 0.02 | -2.5 ± 0.3 | | 114414 | 12 | | 55/45 | The Vapor Girl Inc. |
| 82 | Chocolate Fudge | | 0.66 | -0.18 ± 0.03 | 0.18 ± 0.01 | 0.99 ± 0.01 | 0.81 ± 0.02 | -1.9 ± 0.2 | | 114414 | 12 | | 55/45 | The Vapor Girl Inc. |
| 74 | Chocolate Moo | | 0.72 | -0.14 ± 0.03 | 0.17 ± 0.01 | 0.99 ± 0.01 | 0.82 ± 0.02 | -2.0 ± 0.2 | | 114414 | 12 | | 55/45 | The Vapor Girl Inc. |
| 95 | Chocolate Pecan Fudge | | 0.56 | -0.26 ± 0.04 | 0.07 ± 0.02 | 1.04 ± 0.02 | 0.97 ± 0.02 | -3.0 ± 0.5 | | 114414 | 12 | | 55/45 | The Vapor Girl Inc. |
| 126 | Chocolate Tobacco Heaven | | 1.22 | 0.08 ± 0.03 | 0.10 ± 0.02 | 1.10 ± 0.01 | 1.00 ± 0.02 | -2.5 ± 0.3 | | 114414 | 12 | | 55/45 | The Vapor Girl Inc. |
| 15 | Cinnamon Roll | | 0.68 | -0.17 ± 0.05 | 0.18 ± 0.02 | 1.12 ± 0.02 | 0.95 ± 0.03 | -2.3 ± 0.4 | | 114414 | 12 | | 55/45 | The Vapor Girl Inc. |
| 75 | Circus Guava | | 1.23 | 0.09 ± 0.03 | 0.10 ± 0.02 | 1.05 ± 0.01 | 0.95 ± 0.02 | -3.4 ± 0.7 | | 114414 | 12 | | 55/45 | The Vapor Girl Inc. |
| 163 | City of Angels | | 2.35 | 0.37 ± 0.03 | 0.22 ± 0.02 | 1.01 ± 0.02 | 0.79 ± 0.03 | -2.5 ± 0.5 | | L1 | 0 | | 55/45 | E-Tonic |
| 136 | Classic Tobacco | | 3.32 | 0.52 ± 0.02 | 0.12 ± 0.03 | 1.07 ± 0.02 | 0.95 ± 0.04 | -2.6 ± 0.4 | | A4G03 | 10 | | 55/45 | NJOY |
| 123 | Clove Cigar | | 3.26 | 0.51 ± 0.01 | 0.17 ± 0.02 | 1.08 ± 0.01 | 0.91 ± 0.03 | -7.4 ± 2.0 | | 114414 | 12 | | 55/45 | The Vapor Girl Inc. |
| 107 | Coconut Rum | | 3.05 | 0.484 ± 0.009 | 0.18 ± 0.02 | 1.10 ± 0.01 | 0.93 ± 0.02 | -8.0 ± 1.9 | | 114414 | 12 | | 55/45 | The Vapor Girl Inc. |
| 72 | Coconut Water | | 5.54 | 0.74 ± 0.02 | 0.33 ± 0.02 | 1.05 ± 0.01 | 0.72 ± 0.02 | -3.1 ± 0.4 | | 114414 | 12 | | 55/45 | The Vapor Girl Inc. |
| 124 | Cool Mint | | 0.64 | -0.19 ± 0.06 | 0.11 ± 0.02 | 1.11 ± 0.01 | 1.01 ± 0.02 | -4.6 ± 1.3 | | 114414 | 12 | | 55/45 | The Vapor Girl Inc. |
| 108 | Cotton Berry | | 0.58 | -0.24 ± 0.04 | 0.07 ± 0.02 | 1.02 ± 0.02 | 0.95 ± 0.03 | -2.1 ± 0.3 | | 114414 | 12 | | 55/45 | The Vapor Girl Inc. |
| 24 | Crabtastic! | | 3.63 | 0.56 ± 0.02 | 0.42 ± 0.02 | 1.11 ± 0.01 | 0.69 ± 0.02 | -4.3 ± 0.6 | | 114414 | 12 | | 55/45 | The Vapor Girl Inc. |
| 117 | Cranberry Crunch | | 3.47 | 0.54 ± 0.02 | 0.18 ± 0.02 | 1.13 ± 0.01 | 0.95 ± 0.03 | -4.7 ± 1.0 | | 114414 | 12 | | 55/45 | The Vapor Girl Inc. |
| 99 | Cranberry Delight! | | 2.64 | 0.42 ± 0.05 | 0.06 ± 0.04 | 0.89 ± 0.02 | 0.83 ± 0.05 | -1.6 ± 0.3 | | 114414 | 12 | | 55/45 | The Vapor Girl Inc. |
| 121 | Crispy Melon | | 3.66 | 0.56 ± 0.02 | 0.17 ± 0.03 | 1.10 ± 0.01 | 0.93 ± 0.03 | -3.8 ± 0.8 | | 114414 | 12 | | 55/45 | The Vapor Girl Inc. |
| 30 | Cuba Libre | | 0.50 | -0.30 ± 0.05 | 0.31 ± 0.01 | 0.85 ± 0.02 | 0.54 ± 0.02 | -2.2 ± 0.4 | | 114414 | 12 | | 55/45 | The Vapor Girl Inc. |
| 58 | DB's Dessert | | 2.08 | 0.32 ± 0.03 | 0.21 ± 0.02 | 1.12 ± 0.01 | 0.90 ± 0.03 | -2.3 ± 0.3 | | 114414 | 12 | | 55/45 | The Vapor Girl Inc. |
| 28 | Death Flirt | | 2.59 | 0.41 ± 0.02 | 0.26 ± 0.01 | 1.02 ± 0.01 | 0.76 ± 0.02 | -3.1 ± 0.5 | | 114414 | 12 | | 55/45 | The Vapor Girl Inc. |
| 22 | Desert Cow | | 3.70 | 0.57 ± 0.01 | 0.33 ± 0.01 | 1.05 ± 0.01 | 0.72 ± 0.02 | -4.8 ± 0.6 | | 114414 | 12 | | 55/45 | The Vapor Girl Inc. |
| 137 | Double Espresso | | 1.47 | 0.17 ± 0.08 | 0.13 ± 0.05 | 1.03 ± 0.01 | 0.90 ± 0.05 | -1.0 ± 0.1 | | A4F40 | 10 | | 55/45 | NJOY |
| 73 | Dulce de Leche | | 0.64 | -0.19 ± 0.03 | 0.11 ± 0.01 | 1.04 ± 0.01 | 0.93 ± 0.02 | -2.5 ± 0.3 | | 114414 | 12 | | 55/45 | The Vapor Girl Inc. |
| 13 | Energon | | 3.14 | 0.50 ± 0.02 | 0.24 ± 0.01 | 1.10 ± 0.02 | 0.86 ± 0.03 | -2. ± 0.4 | | 114414 | 12 | | 55/45 | The Vapor Girl Inc. |
| 83 | French Vanilla Cinnamon Coffee | | 1.17 | 0.07 ± 0.02 | 0.13 ± 0.01 | 1.05 ± 0.01 | 0.93 ± 0.02 | -2.0 ± 0.2 | | 114414 | 12 | | 55/45 | The Vapor Girl Inc. |
| 86 | French Vanilla Hazelnut Espresso | | 1.42 | 0.15 ± 0.01 | 0.155 ± 0.008 | 1.040 ± 0.006 | 0.88 ± 0.01 | -3.1 ± 0.2 | | 114414 | 12 | | 55/45 | The Vapor Girl Inc. |
| 41 | Fruit Loopy | | 2.12 | 0.33 ± 0.03 | 0.15 ± 0.02 | 1.08 ± 0.01 | 0.93 ± 0.02 | -3.9 ± 0.7 | | 114414 | 12 | | 55/45 | The Vapor Girl Inc. |
| 33 | Georgia Juicy Peach | | 3.91 | 0.59 ± 0.02 | 0.33 ± 0.02 | 1.11 ± 0.01 | 0.78 ± 0.02 | -4.3 ± 0.6 | | 114414 | 12 | | 55/45 | The Vapor Girl Inc. |
| 103 | Grape Soda | | 1.69 | 0.23 ± 0.03 | 0.36 ± 0.02 | 1.07 ± 0.01 | 0.72 ± 0.02 | -1.8 ± 0.2 | | 114414 | 12 | | 55/45 | The Vapor Girl Inc. |
| 32 | Grape! | | 3.71 | 0.57 ± 0.02 | 0.33 ± 0.02 | 1.07 ± 0.01 | 0.74 ± 0.02 | -3.9 ± 0.5 | | 114414 | 12 | | 55/45 | The Vapor Girl Inc. |
| 159 | Green Gummies | | 0.40 | -0.40 ± 0.04 | 0.20 ± 0.02 | 1.08 ± 0.01 | 0.88 ± 0.02 | -2.0 ± 0.3 | | L1 | 0 | | 55/45 | E-Tonic |
| 119 | Honey Vanilla Tobacco | | 1.50 | 0.18 ± 0.02 | 0.10 ± 0.01 | 1.061 ± 0.008 | 0.96 ± 0.01 | -2.8 ± 0.2 | | 114414 | 12 | | 55/45 | The Vapor Girl Inc. |
| 130 | Hot Cinnamon Candies | | 0.42 | -0.37 ± 0.04 | 0.19 ± 0.02 | 1.12 ± 0.01 | 0.93 ± 0.03 | -2.4 ± 0.4 | | 114909 | 0 | | 55/45 | The Vapor Girl Inc. |
| 4 | Hot Cinnamon Candies | | 0.32 | -0.50 ± 0.03 | 0.14 ± 0.02 | 1.05 ± 0.01 | 0.91 ± 0.02 | -1.9 ± 0.2 | | 1523673 | 12 | | 55/45 | The Vapor Girl Inc. |
| 81 | Icy Blast | | 5.04 | 0.70 ± 0.04 | 0.14 ± 0.04 | 1.096 ± 0.008 | 0.95 ± 0.04 | -1.8 ± 0.2 | | 114414 | 12 | | 55/45 | The Vapor Girl Inc. |
| 91 | Key Lime Pie | | 2.97 | 0.47 ± 0.03 | 0.14 ± 0.03 | 1.07 ± 0.01 | 0.92 ± 0.04 | -2.4 ± 0.5 | | 114414 | 12 | | 55/45 | The Vapor Girl Inc. |
| 113 | KIWI Blast | | 4.29 | 0.63 ± 0.03 | 0.14 ± 0.04 | 1.13 ± 0.01 | 0.99 ± 0.04 | -2.3 ± 0.4 | | 114414 | 12 | | 55/45 | The Vapor Girl Inc. |
| 131 | Kola | | 1.17 | 0.07 ± 0.04 | 0.20 ± 0.03 | 1.12 ± 0.01 | 0.91 ± 0.03 | -2.6 ± 0.6 | | 114414 | 12 | | 55/45 | The Vapor Girl Inc. |
| 2 | Kola No Nicotine | | 0.68 | -0.17 ± 0.03 | 0.20 ± 0.02 | 1.09 ± 0.01 | 0.89 ± 0.02 | -3.0 ± 0.5 | | 114909 | 0 | | 55/45 | The Vapor Girl Inc. |
| 110 | Lemon Meringue Pie | | 3.20 | 0.51 ± 0.05 | 0.11 ± 0.05 | 1.13 ± 0.02 | 1.03 ± 0.06 | -2.2 ± 0.6 | | 114414 | 12 | | 55/45 | The Vapor Girl Inc. |
| 17 | Marc's Burro Tobacco | | 2.86 | 0.46 ± 0.02 | 0.21 ± 0.02 | 1.06 ± 0.02 | 0.85 ± 0.03 | -2.8 ± 0.5 | | 114414 | 12 | | 55/45 | The Vapor Girl Inc. |
| 71 | Marshmallow | | 3.06 | 0.49 ± 0.02 | 0.13 ± 0.03 | 1.14 ± 0.01 | 1.02 ± 0.03 | -2.1 ± 0.3 | | 114414 | 12 | | 55/45 | The Vapor Girl Inc. |
| 138 | Menthol | | 0.49 | -0.31 ± 0.04 | 0.20 ± 0.02 | 1.011 ± 0.009 | 0.81 ± 0.02 | -1.7 ± 0.2 | | A4F44 | 10 | | 55/45 | NJOY |
| 10 | Menthol Tobacco | | 3.24 | 0.51 ± 0.06 | 0.08 ± 0.06 | 1.11 ± 0.01 | 1.03 ± 0.06 | -1.5 ± 0.3 | | 114909 | 0 | | 55/45 | The Vapor Girl Inc. |
| 132 | Menthol Tobacco | | 1.38 | 0.14 ± 0.03 | 0.24 ± 0.02 | 1.051 ± 0.009 | 0.81 ± 0.02 | -3.3 ± 0.6 | | 114414 | 12 | | 55/45 | The Vapor Girl Inc. |
| 88 | Missed Her Cookie | | 1.80 | 0.26 ± 0.02 | 0.14 ± 0.01 | 1.013 ± 0.008 | 0.87 ± 0.01 | -2.8 ± 0.2 | | 114414 | 12 | | 55/45 | The Vapor Girl Inc. |
| 160 | Mojito | | 0.51 | -0.29 ± 0.04 | 0.18 ± 0.02 | 1.05 ± 0.01 | 0.87 ± 0.03 | -2.2 ± 0.3 | | L1 | 0 | | 55/45 | E-Tonic |
| 29 | Mt. DUDE | | 2.19 | 0.34 ± 0.04 | 0.24 ± 0.03 | 1.10 **±** 0.01 | 0.87 ± 0.03 | -2.2 ± 0.4 | | 114414 | 12 | | 55/45 | The Vapor Girl Inc. |
| 25 | Orphan Tears | | 2.67 | 0.43 ± 0.02 | 0.38 ± 0.02 | 1.08 ± 0.02 | 0.70 ± 0.02 | -4.5 ± 1.4 | | 114414 | 12 | | 55/45 | The Vapor Girl Inc. |
| 162 | Peach | | 3.29 | 0.52 ± 0.03 | 0.47 ± 0.03 | 1.12 ± 0.03 | 0.65 ± 0.04 | -4.3 ± 1.6 | | L1 | 0 | | 55/45 | E-Tonic |
| 14 | Peach Piano | | 3.27 | 0.51 ± 0.01 | 0.24 ± 0.02 | 1.08 ± 0.01 | 0.84 ± 0.02 | -3.8 ± 0.6 | | 114414 | 12 | | 55/45 | The Vapor Girl Inc. |
| 139 | Peach Tea | | 2.07 | 0.32 ± 0.05 | 0.15 ± 0.04 | 1.15 ± 0.01 | 1.00 ± 0.05 | -1.6 ± 0.3 | | A4F35 | 10 | | 55/45 | NJOY |
| 66 | Peaches N Cream | | 4.64 | 0.67 ± 0.03 | 0.41 ± 0.03 | 1.05 ± 0.02 | 0.64 ± 0.03 | -3.0 ± 0.5 | | 114414 | 12 | | 55/45 | The Vapor Girl Inc. |
| 3 | Peanut Butter Cookies | | 2.33 | 0.37 ± 0.02 | 0.12 ± 0.02 | 1.01 ± 0.01 | 0.89 ± 0.02 | -3.0 ± 0.4 | | 114414 | 12 | | 55/45 | The Vapor Girl Inc. |
| 21 | Pillow Fight | | 3.59 | 0.56 ± 0.02 | 0.37 ± 0.02 | 1.15 ± 0.01 | 0.78 ± 0.02 | -3.3 ± 0.4 | | 114414 | 12 | | 55/45 | The Vapor Girl Inc. |
| 98 | Pixie Dust | | 2.13 | 0.33 ± 0.04 | 0.31 ± 0.02 | 1.05 ± 0.01 | 0.74 ± 0.02 | -3.3 ± 0.7 | | 114414 | 12 | | 55/45 | The Vapor Girl Inc. |
| 134 | Pomegranate | | 4.36 | 0.64 ± 0.05 | 0.10 ± 0.05 | 1.15 ± 0.01 | 1.05 ± 0.05 | -2.7 ± 0.6 | | 4AF34 | 10 | | 55/45 | NJOY |
| 11 | Popcorn | | 3.52 | 0.55 ± 0.03 | 0.17 ± 0.03 | 1.01 ± 0.01 | 0.83 ± 0.04 | -1.6 ± 0.2 | | 114414 | 12 | | 55/45 | The Vapor Girl Inc. |
| 127 | Pumpkin Pie | | 0.47 | -0.33 ± 0.05 | 0.19 ± 0.02 | 1.08 ± 0.01 | 0.89 ± 0.02 | -4.4 ± 1.0 | | 114414 | 12 | | 55/45 | The Vapor Girl Inc. |
| 40 | Raspberry | | 5.25 | 0.72 ± 0.02 | 0.23 ± 0.02 | 1.052 ± 0.009 | 0.82 ± 0.02 | -2.6 ± 0.2 | | 114414 | 12 | | 55/45 | The Vapor Girl Inc. |
| 157 | Red Gummies | | 5.21 | 0.72 ± 0.03 | 0.41 ± 0.03 | 1.14 ± 0.02 | 0.72 ± 0.04 | -3.2 ± 0.6 | | L1 | 0 | | 55/45 | E-Tonic |
| 94 | Root Beer | | 1.66 | 0.22 ± 0.02 | 0.19 ± 0.01 | 1.01 ± 0.01 | 0.82 ± 0.02 | -2.7 ± 0.2 | | 114414 | 12 | | 55/45 | The Vapor Girl Inc. |
| 48 | RY4 Classic | | 3.97 | 0.60 ± 0.02 | 0.26 ± 0.02 | 1.02 ± 0.01 | 0.76 ± 0.02 | -3.0 ± 0.4 | | 114414 | 12 | | 55/45 | The Vapor Girl Inc. |
| 36 | RY4 Doubler | | 3.97 | 0.60 ± 0.02 | 0.19 ± 0.03 | 0.98 ± 0.02 | 0.79 ± 0.03 | -3.0 ± 0.5 | | 114414 | 12 | | 55/45 | The Vapor Girl Inc. |
| 133 | Single Malt Scotch | | 3.15 | 0.50 ± 0.07 | 0.13 ± 0.05 | 1.06 ± 0.01 | 0.93 ± 0.06 | -1.3 ± 0.2 | | 4AF42 | 10 | | 55/45 | NJOY |
| 18 | Slug Juice | | 3.66 | 0.56 ± 0.02 | 0.44 ± 0.02 | 1.14 ± 0.01 | 0.70 ± 0.02 | -3.2 ± 0.4 | | 114414 | 12 | | 55/45 | The Vapor Girl Inc. |
| 5 | Solid Menthol | | 1.95 | 0.29 ± 0.04 | 0.21 ± 0.02 | 0.96 ± 0.02 | 0.75 ± 0.03 | -1.7 ± 0.2 | | 114414 | 12 | | 55/45 | The Vapor Girl Inc. |
| 118 | Sour Fruit Punch | | 4.88 | 0.69 ± 0.06 | 0.02 ± 0.08 | 1.10 ± 0.02 | 1.07 ± 0.09 | -1.6 ± 0.3 | | 114414 | 12 | | 55/45 | The Vapor Girl Inc. |
| 116 | Strawberries and Champagne | | 3.70 | 0.57 ± 0.01 | 0.18 ± 0.02 | 1.04 ± 0.01 | 0.85 ± 0.02 | -2.6 ± 0.2 | | 114414 | 12 | | 55/45 | The Vapor Girl Inc. |
| 70 | Strawberry | | 3.47 | 0.54 ± 0.02 | 0.09 ± 0.03 | 1.04 ± 0.01 | 0.95 ± 0.03 | -2.5 ± 0.3 | | 114414 | 12 | | 55/45 | The Vapor Girl Inc. |
| 35 | Strawberry Mango Smoothie | | 3.68 | 0.57 ± 0.02 | 0.26 ± 0.03 | 1.08 ± 0.01 | 0.82 ± 0.03 | -2.8 ± 0.4 | | 114414 | 12 | | 55/45 | The Vapor Girl Inc. |
| 120 | Strawberry Pops O'Tart | | 1.56 | 0.19 ± 0.02 | 0.102 ± 0.009 | 0.99 ± 0.01 | 0.89 ± 0.01 | -3.6 ± 0.3 | | 114414 | 12 | | 55/45 | The Vapor Girl Inc. |
| 93 | Sugar Cookie | | 0.75 | -0.12 ± 0.04 | 0.07 ± 0.02 | 1.04 ± 0.02 | 0.97 ± 0.03 | -2.7 ± 0.5 | | 114414 | 12 | | 55/45 | The Vapor Girl Inc. |
| 77 | Sweet Potato Pie | | 3.18 | 0.50 ± 0.02 | 0.21 ± 0.02 | 1.054 ± 0.008 | 0.85 ± 0.02 | -2.5 ± 0.3 | | 114414 | 12 | | 55/45 | The Vapor Girl Inc. |
| 92 | Tiramisu | | 1.86 | 0.27 ± 0.01 | 0.131 ± 0.009 | 1.020 ± 0.008 | 0.89 ± 0.01 | -2.8 ± 0.2 | | 114414 | 12 | | 55/45 | The Vapor Girl Inc. |
| 38 | Trip in the Woods | | 1.81 | 0.3 ± 0.1 | 0.39 ± 0.05 | 1.02 ± 0.04 | 0.64 ± 0.07 | -1.9 ± 0.8 | | 114414 | 12 | | 55/45 | The Vapor Girl Inc. |
| 31 | Valkyrie | | 3.07 | 0.49 ± 0.03 | 0.11 ± 0.03 | 1.07 ± 0.01 | 0.96 ± 0.03 | -2.0 ± 0.3 | | 114414 | 12 | | 55/45 | The Vapor Girl Inc. |
| 140 | Vanilla Bean | | 0.16 | -0.81 ± 0.06 | 0.05 ± 0.02 | 0.94 ± 0.03 | 0.88 ± 0.04 | -1.4 ± 0.3 | | A4F33 | 10 | | 55/45 | NJOY |
| 312 | Vanilla Bean 2 | | 1.07 | 0.03 ± 0.04 | 0.14 ± 0.04 | 1.16 ± 0.02 | 1.02 ± 0.06 | -2.6 ± 0.9 | | A5604 | 10 | | 55/45 | NJOY |
| 51 | Vanilla Custard | | 0.75 | -0.12 ± 0.03 | 0.17 ± 0.01 | 0.99 ± 0.01 | 0.82 ± 0.02 | -1.7 ± 0.1 | | 114414 | 12 | | 55/45 | The Vapor Girl Inc. |
| 8 | Vanilla Tobacco | | 1.34 | 0.13 ± 0.02 | 0.09 ± 0.01 | 0.99 ± 0.01 | 0.90 ± 0.02 | -3.0 ± 0.3 | | 114414 | 12 | | 55/45 | The Vapor Girl Inc. |
| 46 | Watermelon | | 4.34 | 0.64 ± 0.02 | 0.24 ± 0.03 | 1.05 ± 0.01 | 0.81 ± 0.03 | -2.3 ± 0.3 | | 114414 | 12 | | 55/45 | The Vapor Girl Inc. |
| 161 | White Gummies | | 4.04 | 0.61 ± 0.06 | 0.45 ± 0.04 | 0.88 ± 0.01 | 0.43 ± 0.04 | -3.2 ± 1.2 | | L1 | 0 | | 55/45 | E-Tonic |
| 52 | Wintergreen | | 3.37 | 0.53 ± 0.01 | 0.29 ± 0.02 | 1.06 ± 0.01 | 0.77 ± 0.02 | -5.1 ± 0.8 | | 114414 | 12 | | 55/45 | The Vapor Girl Inc. |
| 164 | 55:45 PGVG | | 5.56 | 0.745 ± 0.008 | 0.256 ± 0.009 | 1.054 ± 0.00.5 | 0.80 ± 0.01 | -2.6 ± 0.1 | | N/A | 0 | | 55/45 | Sigma-Aldrich |
