# Supplementary material for: Evaluation of e-liquid toxicity using an open-source high-throughput screening assay
Source: PLoS Biol. 2018 Mar 27;16(3):e2003904. doi: 10.1371/journal.pbio.2003904 (PMC5870948; doi:10.1371/journal.pbio.2003904)
Supplement: S2 Table — (DOCX) [file pbio.2003904.s002.docx]

**S2 Table.** Sample preparation for e-liquid quantification.

| **E-liquid** | **Dilution Factor** | **Carrier Gas Split Ratio** | **Flavor Quantified** | **Spiked Concentrations (µM)** |
| --- | --- | --- | --- | --- |
| Bahama Mama | 100 | 1:10 | Vanillin | 0, 50, 100, 125*, 150, 200, 250, 300 |
| Banana Pudding | 1000 | 1:25 | Vanillin | 0, 50, 100, 125*, 150, 200, 250, 300 |
| Chocolate Banana | 1000 | 1:25 | Vanillin | 0, 50, 100, 125*, 150, 200, 250, 300 |
| Sour Fruit Punch | 100 | 1:10 | Triacetin | 0, 5, 10, 20, 25*, 30, 40, 50 |
| Kola (-N) | 100 | 1:10 | Triacetin | 0, 5, 10, 20, 25*, 30, 40, 50 |
| Apple Pie | 100 | 1:10 | Triacetin | 0, 10, 20, 25*, 30, 40, 50 |
| Red Gummies | 5000 | 1:100 | Triacetin | 0, 20, 40, 50*, 60, 80, 100 |
| Mojito | 5000 | 1:100 | Triacetin | 0, 20, 40, 50*, 60, 80, 100 |
| Peach | 5000 | 1:100 | Triacetin | 0, 20, 40, 50*, 60, 80, 100 |
| City of Angels | 5000 | 1:100 | Triacetin | 0, 20, 40, 50*, 60, 80, 100 |
| Hot Cinnamon Candies | 1000 | N/A | Cinnamaldehyde | 0,25,50,60*,75,100 |
| Cinnamon Roll | 1000 | N/A | Cinnamaldehyde | 0,25,50,60*,75,100 |
| French Vanilla Cinnamon Coffee | 1000 | N/A | Cinnamaldehyde | 0,25,50,60*,75,100 |
| Root Beer | 1000 | N/A | Cinnamaldehyde | 0,25,50,60*,75,100 |
| Blueberry Cinnamon-Streusal Muffin | 1000 | N/A | Cinnamaldehyde | 0,25,50,60*,75,100 |
| Pumpkin Pie | 1000 | N/A | Cinnamaldehyde | 0,25,50,60*,75,100 |
| Hot Cinnamon Candies (no nicotine) | 1000 | N/A | Cinnamaldehyde | 0,25,50,60*,75,100 |

*Spike used for quality control of quantification method.
