# Supplementary material for: Evaluation of e-liquid toxicity using an open-source high-throughput screening assay
Source: PLoS Biol. 2018 Mar 27;16(3):e2003904. doi: 10.1371/journal.pbio.2003904 (PMC5870948; doi:10.1371/journal.pbio.2003904)
Supplement: S3 Table — SIM, selected ion monitoring. (DOCX) [file pbio.2003904.s003.docx]

**S3 Table.** Selected ion monitoring (SIM) parameters used for quantification of e-liquid flavors.

| **Flavor** | **Quantitative Ion (*m/z*)** | **Qualitative Ions (*m/z*)** |
| --- | --- | --- |
| Triacetin | 43.2 | 103.1, 145.1 |
| Vanillin | 151.1 | 123.2, 81.0 |
| Cinnamaldehyde | 158.9 | 145.0, 186.9 |
